# Transcriptomic profiling of thyroid eye disease orbital fibroblasts identifies sorafenib as a novel therapeutic

**DOI:** 10.3389/fendo.2026.1870087

**Published:** 2026-08-19

**Authors:** Kyle Yuan, Phillip Truong, Charkira C. Patrick, Emma Ushchak, Elisa Roztocil, Steven E. Feldon, Collynn F. Woeller

**Affiliations:** 1 University of Rochester School of Medicine and Dentistry, Rochester, NY, United States; 2 Flaum Eye Institute, University of Rochester Medical Center, Rochester, NY, United States; 3 Department of Pathology, University of Rochester Medical Center, Rochester, NY, United States; 4 Department of Microbiology and Immunology, University of Rochester Medical Center, Rochester, NY, United States; 5 Center for Visual Sciences, University of Rochester Medical Center, Rochester, NY, United States; 6 Department of Environmental Medicine, University of Rochester Medical Center, Rochester, NY, United States

**Keywords:** angiogenesis, fibroblast, Graves’ orbitopathy, PI3K/AKT, RNA-Seq, sorafenib, thyroid eye disease

## Abstract

**Introduction:**

Thyroid eye disease (TED) is a debilitating condition characterized by orbital fibroblast (OF) activation and excessive hyaluronan (HA) accumulation within the retroocular space. While IGF-1R blockade has significantly advanced TED management, incomplete clinical responses and disease relapse underscore the need to identify alternative targets.

**Methods:**

We performed high-throughput RNA sequencing on primary human TED OFs compared with non-TED controls, followed by pathway enrichment analysis and confirmation of select targets by RT-qPCR. We also performed computational drug repositioning using the TED OF gene signature. Candidate therapeutic activity was validated using functional assays measuring AKT phosphorylation, gene expression, and HA synthesis in primary TED OFs.

**Results:**

Our analysis identified enrichment of pathways critical to the TED phenotype, including PI3K-AKT signaling, the platelet-derived growth factor (PDGF) pathway, and extracellular matrix remodeling. We validated several key upregulated mediators that may contribute to orbital remodeling, including FOXC2, HGF, MET, HMGA2, and PLEKHG5, alongside the downregulation of the Wnt antagonist SFRP2. We identified sorafenib, which targets VEGFR, PDGFR, and RAF, as a leading candidate to counteract the TED-specific gene signature. Functional assays demonstrated that sorafenib dose-dependently inhibited PDGFβ-induced AKT phosphorylation, gene expression, and significantly attenuated PDGFβ-induced HA synthesis in primary TED OFs.

**Conclusions:**

These results define a persistent, receptor tyrosine kinase-driven program in orbital fibroblasts obtained from TED and provide a molecular rationale for repurposing sorafenib as a therapeutic strategy for patients with refractory or relapsing TED.

## Highlights

- Thyroid eye disease (TED) orbital fibroblasts exhibit a transcriptomic signature characterized by elevated PI3K/AKT, angiogenic, and growth factor signaling.
- Computational drug prediction identifies sorafenib as a candidate for TED.
- Sorafenib dose-dependently inhibits PDGF-driven AKT activation, gene expression, and hyaluronan production in TED orbital fibroblasts.
- Clinical significance: While inhibition of the insulin-like growth factor-1 receptor (IGF-1R) has advanced the management of thyroid eye disease (TED), some patients remain refractory or relapse after treatment ceases. Our study identifies a persistent growth factor signaling program in TED orbital fibroblasts and demonstrates that sorafenib suppresses PDGF-induced expression of genes within the pathogenic signature. These findings establish a molecular rationale for repurposing sorafenib to treat refractory or relapsing TED.

## Introduction

Thyroid eye disease (TED) is a debilitating autoimmune condition characterized by expansion of orbital adipose tissue and fibrosis of the extraocular muscles, leading to proptosis, diplopia, and vision loss. The annual incidence of TED is 2 in every 10,000 individuals and is highly associated with Graves’ disease (GD): 25%-40% of individuals with GD are diagnosed with TED (1, 2). Among those, moderate-to-severe TED affects an estimated 4.4-9.0 per 100,000 individuals, with women disproportionately affected (3). Treatments for TED include systemic glucocorticoids, mycophenolate, and orbital decompression surgery (4–6), with systemic glucocorticoids as the mainstay of treatment (7–9). Teprotumumab, an insulin-like growth factor-1 receptor (IGF-1R) blocking antibody, has shown great promise, with approximately 80% of patients experiencing a reduction in symptomatic disease (10). Clinical trials have demonstrated significant improvements in key clinical outcomes, including reductions in proptosis (≥2 mm), the Clinical Activity Score (CAS), diplopia, and quality-of-life scores (10, 11).

Despite these benefits, teprotumumab can cause clinically significant adverse events, including hearing loss, muscle spasms, inflammatory bowel disease flares, and hyperglycemia. In a study of 131 patients who received ≥4 infusions, 8.4% experienced severe adverse events and 12.2% discontinued therapy (12). In addition, 20%-30% of TED patients are refractory to first-line treatments, and 10%-20% relapse after treatment is withdrawn (7). The limited therapeutic options and variable response profiles have prompted the use of genomic approaches, such as RNA sequencing (RNA-seq), to better define TED pathogenesis at the molecular level.

Orbital fibroblasts (OFs) are central to TED pathogenesis because they can differentiate into adipocytes or myofibroblasts, secrete large amounts of hyaluronan (HA), and proliferate excessively (2, 13–15). TED OFs express both the thyroid-stimulating hormone receptor (TSHR) and IGF-1R (15–19). Stimulatory TSHR autoantibodies found in patients with TED/Graves’ disease can promote adipogenesis, inflammation, and HA synthesis (20, 21). TSHR and IGF-1R converge on the phosphatidylinositol 3-kinase (PI3K)/AKT pathway, which is increasingly recognized as a nodal regulator of proliferation, cytokine production, and extracellular matrix (ECM) remodeling in TED (15, 22, 23). *In vivo*, TED OFs are also exposed to inflammatory mediators and additional receptor tyrosine kinase (RTK) ligands, including PDGF, VEGF, and TGFβ, which can further engage the PI3K/AKT axis and contribute to orbital pathology (24–28). The complex, dynamic interplay of these signals may explain the clinical heterogeneity observed in TED, in which some patients exhibit predominantly fat expansion, others extraocular muscle enlargement, and many present with a heterogeneous phenotype characterized by increases in both fat and muscle.

To identify new therapeutic targets in TED and given that current animal models only partially reproduce the orbital phenotype (29–32), we utilized primary OFs from orbital fat decompression surgery, a physiologically relevant and widely validated system for dissecting TED-specific signaling (14, 33). Here, we performed transcriptome-wide expression profiling of primary TED and control OFs and applied gene ontology tools, including Enrichr (34–36), ToppGene (37–39), and DAVID (40, 41) to define dysregulated pathways in TED. We then used the LINCS L1000CDS² platform (42) to screen for small molecules predicted to counter the TED OF gene expression signature. This integrative strategy identified sorafenib, a multi-kinase inhibitor targeting RAF, VEGFR, and PDGFR, which we validated functionally in PDGFβ-stimulated TED OFs. Sorafenib dose-dependently suppressed TED-specific gene expression and inhibited HA production, demonstrating its therapeutic potential in TED.

## Methods

### Tissue procurement and study approval

*OFs* were isolated from orbital fat specimens obtained during decompression surgery from patients with thyroid eye disease (TED) (n = 7) or from normal orbital tissue obtained as surgical discard from patients undergoing orbital bone fracture repair (orbitotomy for non-inflammatory conditions such as blow-out fracture), with indications unrelated to orbital pathology and no history of TED, autoimmune thyroid disease, or orbital inflammation (n = 4). Written informed consent was obtained from all participants. Donor demographics are summarized in **Table 1** (additional information on donor strains is listed in **Supplementary Table 3**). Primary cultures were established from fresh tissue using explant outgrowth. Tissue fragments (~1 mm³) were placed in 35-mm dishes containing Dulbecco’s Modified Eagle Medium/Nutrient Mixture F-12 (DMEM/F12; Gibco) supplemented with 10% fetal bovine serum (FBS; Sigma), 2 mM L-glutamine, and antibiotic-antimycotic (100 U/mL penicillin, 100 μg/mL streptomycin, 0.25 μg/mL amphotericin B; Gibco). Cultures were maintained at 37 °C in a humidified 5% CO_2_ incubator. Fibroblasts migrating from explants were expanded to confluence (approximately 2–3 weeks), tissue remnants were removed, and adherent cells were passaged using 0.05% trypsin-EDTA (Gibco). Cell identity was verified by vimentin^+^/cytokeratin^-^/CD45^-^. Cells used for RNA sequencing were all at passage 5; functional experiments used cells between passages 4 and 10.

**Table 1:** Demographic Information for non-TED vs. TED Subjects.

Category	Non-TED	TED
Age (Mean) range (yrs)	(55.0) 45–61	(52.0) 27–62
Smokers (N)	1	4
Steroid Use (N)	1	3
Sex (M, F)	3, 1	1, 6
Total	4	7

### RNA isolation and sequencing

For RNA-seq, total RNA was extracted using the RNeasy Mini Kit (Qiagen) with on-column DNasedigestion (RNase-Free DNase Set, Qiagen). RNA quantity and purity were assessed by NanoDrop spectrophotometry, and integrity by Agilent 2100 Bioanalyzer; only samples with RIN > 8.0 were used. Libraries were prepared with the TruSeq Stranded mRNA Library Prep Kit (Illumina) at the University of Rochester Functional Genomics Core and sequenced single-end (75 bp) on an Illumina NextSeq 2000, with base calls demultiplexed to FASTQ using bcl2fastq 2.19.1; samples yielded a mean of 32.6 million reads (range 28.2 to 35.8 million). Per-sample sequencing and alignment quality metrics are provided in **Supplementary Table 4**.

### Bioinformatics and differential expression analysis

Reads were quality- and adapter-trimmed with fastp (0.23.1) and aligned to the human reference genome (GRCh38.p13, GENCODE release 42) using STAR (2.7.9a); the mean uniquely mapped rate was 92.4% (range 91.4 to 93.3%). Gene-level counts were summarized with featureCounts (Subread 2.0.1) in reverse-stranded mode (-s 2; a mean of 71.4% of reads were assigned to genes, range 68.9 to 74.7%). Reads were processed and analyzed with DESeq2 in R (4.1.1), which models raw counts and applies median-of-ratios normalization. Differential expression between TED and non-TED OFs was assessed with Benjamini–Hochberg correction; adjusted p < 0.05 was considered significant. Genes with no counts in any sample were removed prior to testing. In total, 31,390 genes were tested, and 17,489 retained adjusted p-values after DESeq2 independent filtering. For visualization and unsupervised analyses, normalized counts were transformed with the variance-stabilizing transformation (VST). Gene ontology (GO) and pathway enrichment were performed using Enrichr, ToppGene, and DAVID. KEGG and Reactome were the primary pathway databases queried. Over-representation analyses were performed against each tool’s default background (the annotated human genome), and the results are reported for all platforms. Because over-representation analysis identifies enriched gene sets but does not establish directional pathway activation or inhibition, results are described as enriched rather than activated. KEGG and GO terms are reported at Benjamini-Hochberg adjusted p < 0.05; Reactome terms are reported at FDR < 0.10 and are considered exploratory.

*LINCS Analysis.* To identify compounds predicted to reverse the TED transcriptionalsignature, the significantly upregulated (n = 233) and downregulated (n = 129) genes from the TED vs. non-TED differential expression analysis (adjusted p < 0.05) were queried against the Library of Integrated Network-Based Cellular Signatures (LINCS) L1000 database using the characteristic direction signature search engine (L1000CDS²) run in reverse-signature-search mode to identify molecules predicted to oppose the input gene expression signature. Compounds were ranked by connectivity score (1 − cos α), with higher scores indicating stronger predicted reversal of the TED signature. The top 50-ranked signatures were retained for annotation; uncharacterized probe compounds were excluded, and the complete ranked output is provided in **Supplementary Table 2**. A subset of annotated candidates is summarized in **Table 2**.

**Table 2:** Compounds predicted to oppose the TED orbital fibroblast transcriptional signature (LINCS L1000CDS²).

Rank	Score (1 − cos α)	Perturbation	Target	Mechanism of action
1	1.5737	Brefeldin A	Protein transport	ER/Golgi transport inhibitor
5	1.5112	NVP-AEW541	IGF-1R	IGF-1R inhibitor
8	1.5038	TPCA-1	IKK-2	IKK-2 inhibitor
9	1.4989	Trametinib	MEK1 / MEK2	MEK1/2 inhibitor
10	1.4977	VX-680	Aurora kinase	Aurora kinase inhibitor
11	1.4966	Afatinib	EGFR / HER2	EGFR/HER2 inhibitor
12	1.4951	HY-10992	Chk1 / Chk2	Checkpoint kinase inhibitor
14	1.4929	CPT-11	Topoisomerase I	Topoisomerase I inhibitor
**15**	**1.4925**	**Sorafenib**	**RAF, VEGFR, PDGFR, RET**	**Multi-kinase inhibitor**
16	1.4915	Dasatinib	BCR-ABL, SRC, PDGFR, KIT	Multi-kinase tyrosine inhibitor
17	1.4889	TG101348	JAK2	JAK2 inhibitor
19	1.4841	NVP-TAE684	ALK	ALK inhibitor
22	1.4770	GSK-1070916	Aurora B / Aurora C	Aurora kinase inhibitor
25	1.4747	Tpl2 (COT) inhibitor	MAP3K8	Tpl2 kinase inhibitor
28	1.4724	Geldanamycin	HSP90	HSP90 inhibitor
29	1.4723	Selumetinib	MEK1 / MEK2	MEK1/2 inhibitor
33	1.4686	Ibrutinib	BTK	BTK inhibitor
37	1.4654	Dovitinib	FGFR, VEGFR, PDGFR	Multi-kinase tyrosine inhibitor
39	1.4641	AZD-7762	Chk1	CHK1 inhibitor
44	1.4627	Ethacrynic acid	NKCC2 co-transporter	Loop diuretic
46	1.4619	PD-0325901	MEK1 / MEK2	MEK1/2 inhibitor
47	1.4597	Canertinib	EGFR	EGFR inhibitor

233 up-regulated and 129 down-regulated genes (adjusted p < 0.05) were queried against the L1000CDS² characteristic-direction signature search engine. Molecules are ranked by connectivity score; higher scores indicate a stronger predicted reversal of the input signature. Annotated compounds from the top 50 are shown; uncharacterized probe compounds are omitted for clarity, and the complete ranked output is provided in **Supplementary Table 2**. Scores across the top 50 span a narrow range (1.4591 to 1.5737). Sorafenib (highlighted) ranked 15th (1.4925), within 5% of the top-scoring hit. Sorafenib was selected for functional validation based on target-pathway coverage and clinical availability. Bold indicates sorafenib, the compound selected for functional validation in this study.

### PDGFβ and sorafenib treatment of orbital fibroblasts

For stimulation experiments, OFs at ~70% confluence were washed twice with PBS and placed in DMEM/F12 containing 0.1% FBS with 50 ng/mL recombinant human PDGF-BB (hereafter referred to as PDGFβ) (PeproTech, Cranbury, NJ, USA) for 48 hours at 37 °C, 5% CO_2_. Vehicle controls received the corresponding PDGFβ diluent (0.1% BSA in PBS). Conditioned media were collected for HA ELISA, and cells were harvested for Western blotting and RNA isolation as indicated below. Sorafenib (BAY-43-9006; MedChemExpress) was dissolved in DMSO to prepare stock solutions, which were diluted into 0.1% FBS DMEM/F12. Cells were pre-treated with sorafenib for 30 minutes, after which PDGFβ was added without media change.

*Hyaluronan (HA) ELISA.* HA in conditioned media was quantified using the HA ELISA kit (Echelon Biosciences, Cat. K-1200) according to the manufacturer’s instructions. The media were centrifuged at 300 × g for 5 min to remove debris and then diluted 1:5 in assay buffer. A standard curve (1 MDa HA; 0–5,000 ng/mL) was run in parallel, and samples were assayed in triplicate. Briefly, samples or standards were first incubated with the kit HA detector (biotinylated HA-binding protein) to allow competitive binding of HA. The mixtures were then transferred to the supplied HA ELISA plate, incubated, and washed as instructed. They were then incubated for 30 min with the kit’s streptavidin–alkaline phosphatase reagent. After three washes, plates were developed with 100 μL p-nitrophenyl phosphate (PNPP) for 25 min. Reactions were stopped, and absorbance was read at 405 nm (reference 610 nm) on a Bio-Rad iMark microplate reader. HA concentrations were interpolated from the standard curve.

*Western Blotting.* Cells were washed with ice-cold PBS and then lysed in 50 mM Tris-HCl (pH 6.8), 2% SDS, supplemented with protease and phosphatase inhibitor cocktails (Cell Signaling Technology). Lysates were heated at 95 °C for 5 min, sonicated briefly, and then clarified (14,000 × g, 10 min, 4 °C). Protein concentration was determined using the DC Protein Assay (Bio-Rad). Equal amounts (5–10 µg) were separated on 4–20% Mini-PROTEAN TGX Stain-Free gels (Bio-Rad) and transferred to 0.45 µm Immobilon-PVDF (Millipore). Membranes were blocked in 5% non-fat dry milk/0.1% Tween-20 in TBS (TBS-T) for 1 h and incubated overnight (4 °C) with primary antibodies: thymidylate synthase (TYMS; rabbit, CST #3766, 1:1,000), phospho-AKT (Ser473; rabbit, CST #4060, 1:1,000), total AKT (rabbit, CST #9272, 1:1,000), and β-actin (anti-actin-hFAB Rhodamine, Bio-Rad #12004163, 1:5,000). After TBS-T washes, membranes probed for TYMS, pAKT, and AKT were incubated for 1 h with HRP-conjugated anti-rabbit secondary antibody (Jackson ImmunoResearch, 1:5,000), developed with Immobilon Western Chemiluminescent HRP Substrate (Millipore), and imaged on a ChemiDoc MP system (Bio-Rad). β-actin was detected by direct fluorescence (rhodamine channel) on the same system. Band intensities were quantified with Image Lab software (Bio-Rad). Equal loading was verified by actin and stain-free total protein imaging.

### Real-time quantitative PCR

Total RNA was extracted with QIAzol Lysis Reagent and purified using the RNeasy Mini Kit (Qiagen) according to the manufacturer’s protocol. RNA concentration and purity (A260/A280) were measured on a DS-11+ spectrophotometer (DeNovix). cDNA was synthesized from 500 ng RNA using iScript™ Reverse Transcription Supermix (Bio-Rad). qPCR was performed on a CFX Connect™ Real-Time PCR Detection System (Bio-Rad) using SsoFast™ EvaGreen Supermix (Bio-Rad) and gene-specific primers. Target genes were *FOXC2, HGF, HMGA2, SFRP2, PLEKHG5, MET*, and *HAS2*. Relative mRNA expression was calculated by the 2^^-ΔΔCq^ method, normalized to the geometric mean of *18S rRNA* and *TBP* Cq values. Primer sequences are provided in **Table 3**. Gene stability was evaluated across all donor strains and treatment conditions using geNorm (43); 18S rRNA and TBP showed acceptable stability (pairwise M = 0.93, below the 1.5 threshold), and target expression was normalized to their geometric mean. Technical replicates were averaged within each strain so that each biological donor contributed a single observation (n = 5 independent TED orbital fibroblast strains). Because each strain received all treatment conditions, group differences were assessed by repeated-measures one-way ANOVA with donor strain as the matching factor, followed by Dunnett’s multiple comparisons test. Individual donor values are displayed. Significance is denoted *p < 0.05, **p < 0.01, ***p < 0.001.

**Table 3:** Gene primer sequences used for RT-qPCR.

Gene	Forward primer (5′ - 3′)	Reverse primer (5′ - 3′)
18S rRNA	CTGAGAAACGGCTACCACATC	GCCTCGAAAGAGTCCTGTATTG
TBP	CGAAACGCCGAATATAATCCC	CCCAACTTCTGTACAACTCTAGC
FOXC2	GAGATGTTCAACTCCCACCG	GTATTTCGTGCAGTCGTAGGAG
PLEKHG5	ACGGATCTGCTGTTGGTG	GCACTGTGAAACTCATTCAGG
HMGA2	AACAAGAGTCCCTCTAAAGCAG	GATGTCTCTTCAGTTTCCTCCTG
HGF	GCTATACTCTTGACCCTCACAC	GTAGCCTTCTCCTTGACCTTG
MET	GACTCCTACAACCCGAATACT	ATAGTGCTCCCCAATGAAAGTAG
SFRP2	GACAACGACATAATGGAAACGC	CACACCGTTCAGCTTGTAAATG
HAS2	GCCTCATCTGTGGAGATGGT	ATGCACTGAACACACCCAAA

*Statistics.* HA ELISA, Western blot densitometry, and RT-qPCR data were analyzed in GraphPad Prism (v10). A two-tailed unpaired Student’s *t-test* was used for comparisons between two groups. For comparisons with ≥3 groups, one-way ANOVA followed by Dunnett's *post hoc* test was used; p < 0.05 was considered significant. Sorafenib IC_50_ values were calculated from normalized HA levels using a four-parameter logistic (4PL) fit in Prism. All *in vitro* experiments were performed in at least three independent TED orbital fibroblast strains. The donor-derived strain was the experimental unit: technical triplicates were run per condition. Data are presented as mean ± SEM.

Study approval. This study was performed in accordance with protocols (URMC00099) approved by the IRB at the University of Rochester Medical Center. All participants provided written informed consent.

## Results

### Transcriptomic profiling reveals distinct gene expression signatures in TED orbital fibroblasts

**Figure 1:**
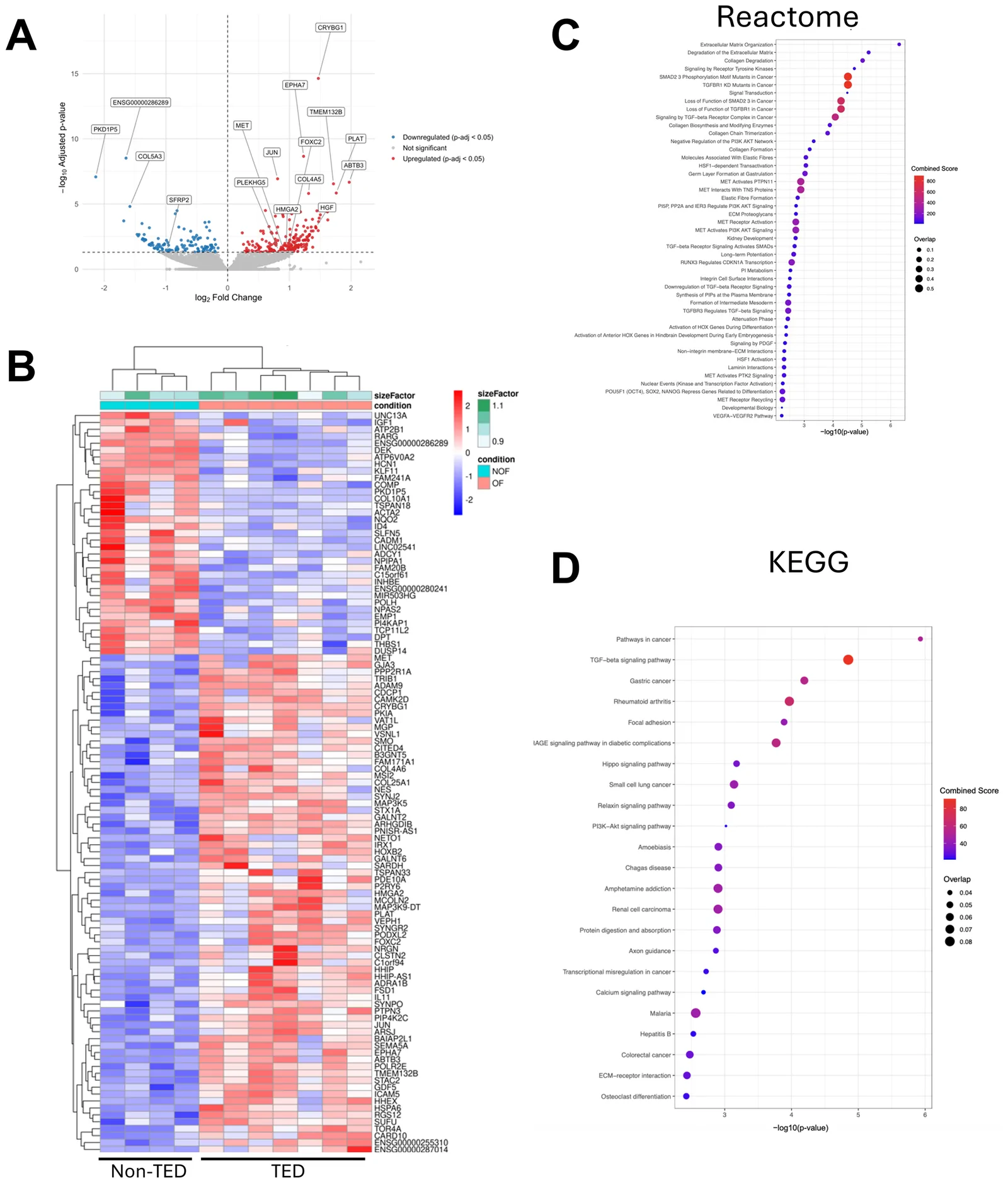
Differential gene expression analysis of TED versus non-TED orbital fibroblasts. **(A)**Volcano plot showing RNA-seq results comparing TED (n = 7) and non-TED (n = 4) orbital fibroblasts.The x-axis represents log_2_ fold change, and the y-axis represents –log_10_ adjusted p-value. Genes meeting adjusted p < 0.05 are highlighted, with upregulated genes in red and downregulated genes in blue. vertical dashed lines indicate │log_2_FC│ = 1 for visual reference only and do not define the differentially expressed gene set. Selected gene names are labeled in the plot. Several of the most significantly altered genes are known regulators of extracellular matrix (ECM) remodeling (FOXC2, COL4A5), growth factor signaling (MET, HGF), and fibroblast activation (JUN, HMGA2, PLEKHG5). **(B)** A heatmap of the top differentially expressed genes, ranked by adjusted p-value, shows 107 differentially expressed genes. The full heatmap is presented in **Supplementary Figure 2**. Expression values are shown in red (upregulated) or blue (downregulated) relative to the group mean, and pathway enrichment analysis of differentially expressed genes in TED OFs is shown. Bubble plots display significantly enriched pathways identified by Enrichr using Reactome **(C)** (p-adj < 0.10, exploratory) and KEGG **(D)** (p-adj < 0.05) databases. Upregulated genes were enriched in extracellular matrix (ECM) organization, collagen metabolism, receptor tyrosine kinase signaling, and TGFβ-related signaling. Bubble size indicates the proportion of input genes in each term; color represents the combined enrichment score.

To define disease-associated transcriptional changes in TED, we performed RNA-seq on OFs from TEDand non-TED donors. To evaluate global transcriptomic structure and the potential influence of donor covariates, we performed unsupervised PCA and hierarchical clustering on the samples (**Supplementary Figure 1**). The largest source of variance was donor sex rather than disease status, a pattern commonly observed in human RNA-seq cohorts and not unexpected given the discordant male and female numbers between the TED and non-TED cohorts (**Table 1**). However, disease status was resolved on the second component (PC2), indicating that TED and non-TED OFs form distinct transcriptional groups separate from the sex effect. Differential expression analysis identified a robust set of upregulated and downregulated genes in TED OFs compared to non-TED controls (**Figure 1A**). Several of the most significantly altered genes are known regulators of extracellular matrix (ECM) remodeling (*FOXC2*, *COL4A5*), growth factor signaling (*MET*, *HGF*), and fibroblast activation (*JUN*, *HMGA2*, *PLEKHG5*). A heatmap of differentially expressed genes revealed that gene expression changes were largely consistent across individual donors (**Figure 1B****;** the full heatmap is shown in **Supplementary Figure 2****).**

### TED OFs are enriched for PI3K/AKT and angiogenic signatures compared to non-TED OFs

A comparison of TED versus non-TED OFs identified 233 upregulated genes and 129 downregulated genes in TED (adjusted p < 0.05). Gene Ontology (GO) analysis revealed activation of *PI3K/AKT signaling* (adjusted p = 0.018). Interestingly, *negative regulation of the PI3K/AKT network* (adjusted p = 0.027) was also significant. Two additional downstream pathways narrowly missed the significance cutoff, including *MET activating PI3K/AKT signaling* (adjusted p = 0.061) and *PI5P, PP2A, and IER3 regulating PI3K/AKT signaling* (adjusted p = 0.061) (**Figures 1C, D**; **Supplementary Table 1**). ToppGene independently identified receptor tyrosine kinase signaling (FDR 0.003) and HGF/MET/PI3K signaling (FDR 0.015) among enriched terms, and DAVID’s PI3K-Akt KEGG term was borderline (FDR 0.06), concordant with the Enrichr-based PI3K/AKT enrichment described above.

TED OFs also showed significant enrichment of angiogenesis-associated pathways. These pathways included: *vascular smooth muscle cell/pericyte differentiation and proliferation* (adjusted p = 0.00008), *extracellular matrix organization* (adjusted p = 0.0004), *positive regulation of smooth muscle cell proliferation* (adjusted p = 0.023), *vascular endothelial cell activation by growth factors* (adjusted p = 0.037), and *sprouting angiogenesis* (adjusted p = 0.039). Upregulation of *SMAD3*, *TGFB1*, and *TGFBR2* contributed to these pathways, suggesting that the TGFβ signaling cascade plays a significant role in regulating angiogenesis (**Supplementary Table 1**). BMP4 and SEMA5A were also upregulated in select angiogenic pathways. BMP4 is a well-established member of the TGFβ superfamily (44), and SEMA5A has previously been associated with angiogenic processes (45).

The ECM, as well as the synthesis and regulation of collagen, play essential roles in angiogenesis by forming a structural scaffold that supports endothelial cell migration (46). Pathways involving the ECM and collagen were significantly upregulated in TED. These pathways included *ECM organization* (adjusted p = 0.0004), *collagen degradation* (adjusted p = 0.002), and *collagen biosynthesis and modifying enzymes* (adjusted p = 0.009). Upregulated genes contributing to these pathways included collagen genes such as COL4A6, COL4A5, COL9A3, COL13A1, and COL25A1, as well as metalloproteinase genes such as ADAMTS8, ADAM9, and MMP1 (**Figures 1C, D**; **Supplementary Table 1****).** Significant pathways were identified among downregulated genes in TED OFs. However, these pathways lacked a consistent functional theme.

**Figure 2:**
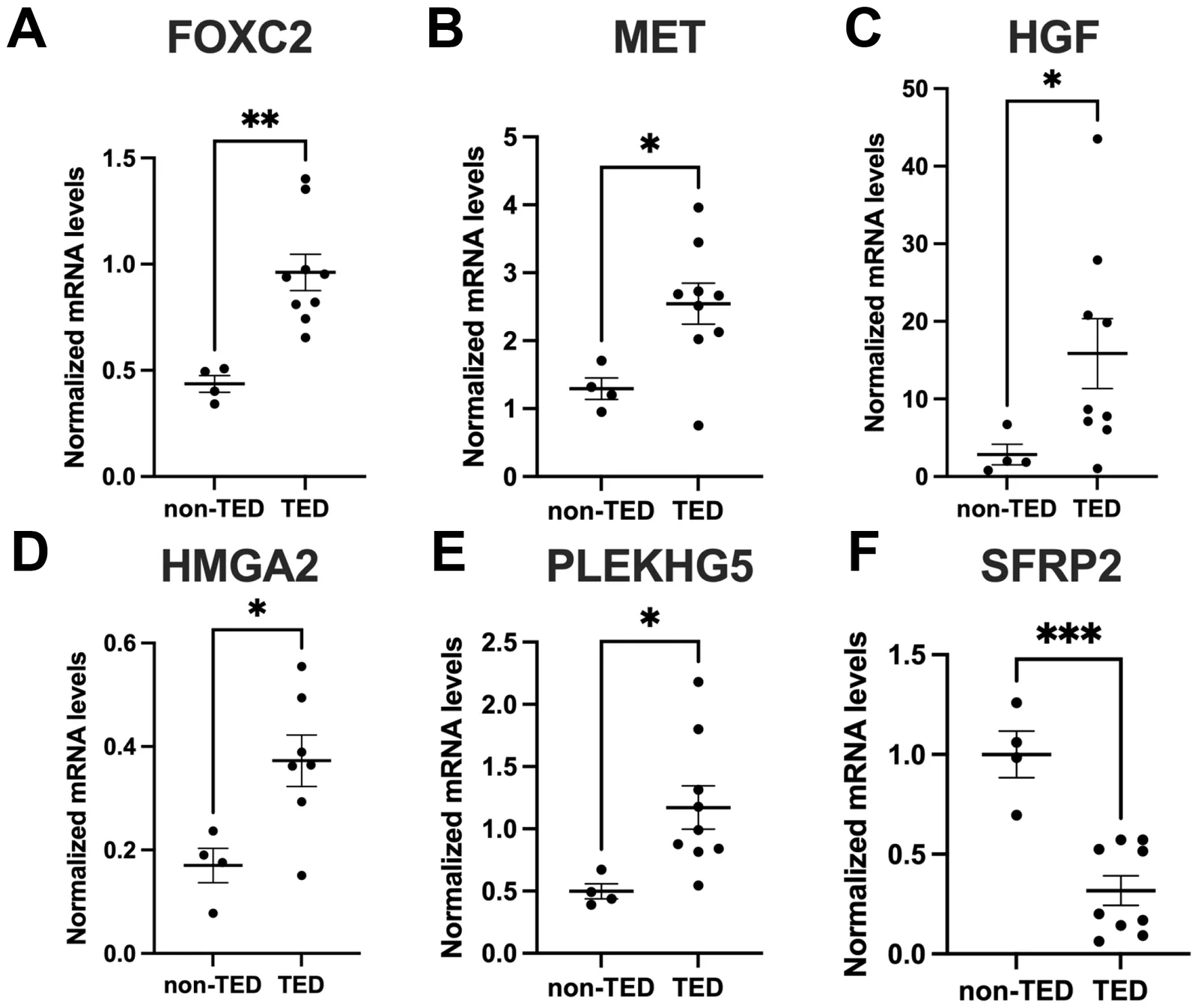
Validation of selected differentially expressed genes by RT-qPCR. Orbital fibroblasts from non-TED (n = 4) and TED (n = 7) donors were analyzed for: (i) angiogenic/RTK-associated genes, *FOXC2, MET, and HGF* [**(A–C)**, respectively]; (ii) fibroblast activation/motility regulators, *HMGA2, and PLEKHG5* **(D, E)**; and (iii) the Wnt antagonist *SFRP2* **(F)**. mRNA levels were normalized to *TBP* and *18S rRNA*. TED OFs showed significantly increased expression of *HGF, MET, FOXC2, HMGA2*, and *PLEKHG5*, and decreased expression of *SFRP2* compared with non-TED OFs. Data are mean ± SEM; *p < 0.05, **p < 0.01, ***p < 0.001 by unpaired two-tailed Student’s t-test.

To validate our RNA-seq results, we performed RT-qPCR on a subset of differentially expressed genes representing the major pathways identified by GO enrichment (PI3K/AKT signaling, angiogenesis, ECM/fibroblast activation) and including genes with large-magnitude, high-confidence changes. First, we assessed pro-angiogenic/RTK-linked genes identified in the transcriptomic analysis (*FOXC2, MET, HGF*). We confirmed that all were significantly higher in TED OFs than in non-TED OFs (**Figures 2A–C**). Second, we measured fibroblast activation/motility-associated transcriptional regulators (*HMGA2, PLEKHG5*), which were also elevated in TED OFs, consistent with an activated fibro-proliferative phenotype (**Figures 2D, E**). Finally, because pathways among downregulated genes lacked a consistent functional theme, we selected *SFRP2*, a negative regulator of Wnt signaling, which was reduced in TED OFs by RNA-seq and confirmed to be significantly downregulated by qPCR (**Figure 2F**). Together, these data validate the RNA-seq findings at the single-gene level and show that TED OFs upregulate angiogenic/RTK and fibroblast activation programs while downregulating the Wnt regulator, *SFRP2*.

### Connectivity map analysis identifies sorafenib as a predicted therapeutic candidate

We next queried the 233 upregulated and 129 downregulated TED-associated genes against the LINCSL1000CDS^2^ characteristic-direction signature search engine to identify compounds predicted to reverse the TED transcriptional signature (42, 47). The top 50-ranked signatures are shown in **Supplementary Table 2**, with annotated candidates summarized in **Table 2**. The computed scores across the top 50 hits were tightly clustered, and this set was dominated by kinase inhibitors, including MEK1/2 inhibitors, receptor tyrosine kinase inhibitors, and aurora kinase inhibitors, a pattern consistent with the RTK- and PI3K/AKT-driven signature identified by our pathway analysis. Notably, the 5th-ranked compound, NVP-AEW541, is a small-molecule IGF-1R inhibitor, the same target as teprotumumab, the only FDA-approved therapy for TED, providing independent support that our L1000CDS² approach recovers pharmacologically validated, disease-relevant targets. Sorafenib, a multi-kinase inhibitor targeting RAF, VEGFR, and PDGFR, ranked 15th overall but was within 5% of the top-scoring compound. Given its coverage of the RTK and angiogenic targets enriched in our transcriptomic data and its established clinical availability, we selected sorafenib for functional validation in primary TED OFs, focusing first on PDGFβ-driven signaling, given its centrality to the angiogenic and RTK pathways identified above.

### Sorafenib suppresses PDGFβ-induced pathogenic gene expression in TED orbital fibroblasts

**Figure 3:**
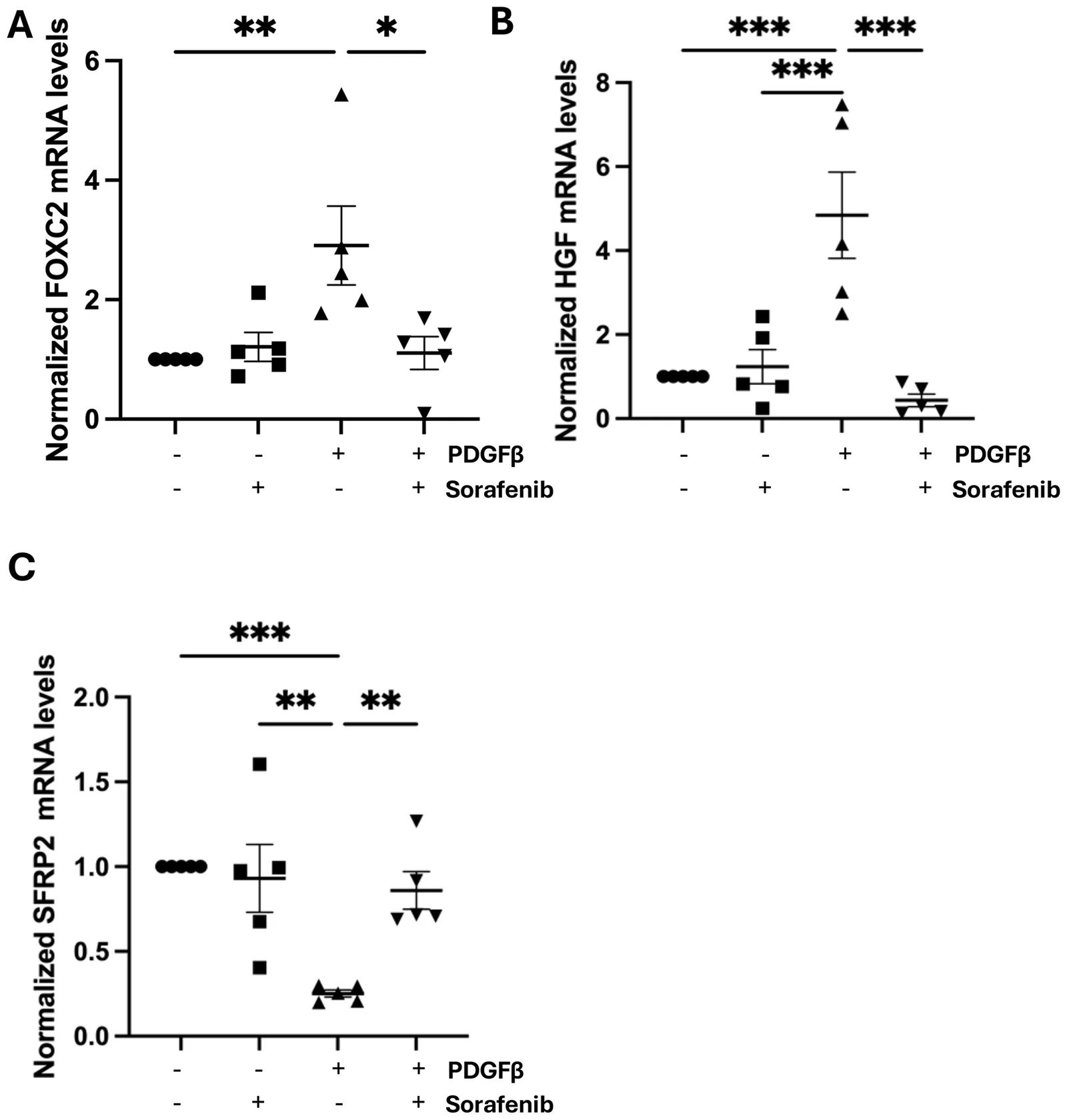
Sorafenib mitigates PDGFβ-induced gene expression in TED orbital fibroblasts. Orbitalfibroblasts from five TED patient-derived strains were treated with vehicle, PDGFβ (50 ng/mL), or PDGFβ + sorafenib (250 nM) for 48 h. mRNA levels of *FOXC2* **(A)** *, HGF* **(B)**, and *SFRP2* **(C)** were measured by RT-qPCR and normalized to *18S rRNA* and *TBP*. PDGFβ significantly increased *FOXC2* and *HGF* expression and reduced *SFRP2*; co-treatment with sorafenib blunted these effects. Each dot represents a single donor strain. Data are mean ± SEM (n = 5 biological donors). Statistical comparisons used repeated-measures one-way ANOVA with Dunnett’s post-test. *p < 0.05, **p < 0.01, ***p < 0.001.

First, we evaluated sorafenib’s ability to block PDGFβ-driven gene expression in TED OFs. We focused on HGF as a representative receptor tyrosine kinase/growth-factor-axis gene, FOXC2 as a representative ECM-remodeling regulator, and SFRP2 as the representative down-regulated Wnt regulator, capturing multiple TED-associated programs and both directions of the sorafenib effect. Treatment with PDGFβ (50 ng/mL) for 48 hours robustly increased mRNA expression of *FOXC2* and *HGF*, while significantly reducing *SFRP2* expression compared to vehicle controls (**Figures 3A–C**). Cotreatment with sorafenib (250 nM) markedly blunted PDGFβ-induced *FOXC2* and *HGF* expression and restored *SFRP2* expression to baseline, indicating a broad inhibitory effect on PDGFβ-mediated transcriptional activation.

**Figure 4:**
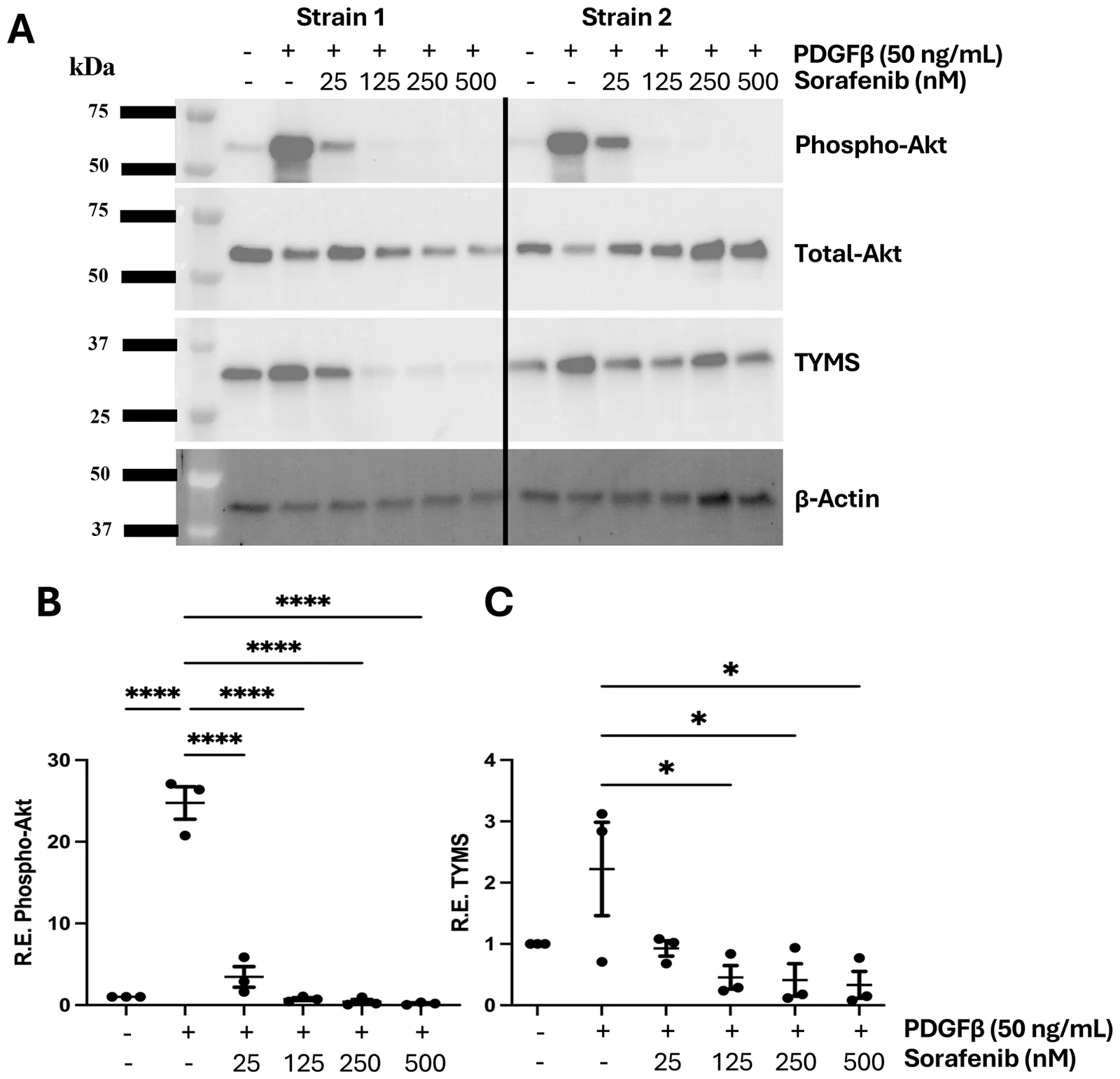
Sorafenib suppresses PDGFβ-induced activation of AKT and thymidylate synthase (TYMS)expression in TED orbital fibroblasts. **(A)** Representative Western blots of TEDfibroblast strains treated with vehicle, PDGFβ (50 ng/mL), or PDGFβ plus increasing sorafenib concentrations (25–500 nM). PDGFβ robustly induced phosphorylation of AKT (phospho-AKT) and increased TYMS expression. Sorafenib reduced phospho-AKT and TYMS in a dose-dependent manner. Total AKT and β-Actin served as loading controls. Uncropped blots are shown in **Supplementary Figures 3–6**. **(B)** Quantification of relative expression (R.E.) of phospho-AKT normalized to total AKT. **(C)** Quantification of TYMS normalized to β-Actin. Data represent mean ± SEM from three independent TED strains (n = 3). *p < 0.05, ****p < 0.0001 by one-way ANOVA.

To determine whether sorafenib’s inhibitory effects on gene expression were associated with attenuation of PDGFβ downstream signaling, we examined activation of AKT and thymidylate synthase (TYMS) expression by Western blot (28). PDGFβ stimulation increased phosphorylation of AKT, while total AKT levels remained unchanged (**Figures 4A, B**). Sorafenib reduced PDGFβ-induced AKT phosphorylation and TYMS expression in a dose-dependent manner, with substantial inhibition observed at concentrations ≥125 nM (**Figures 4B, C**). These findings suggest that sorafenib suppresses PDGFβ-mediated transcriptional responses, in part, by inhibiting AKT signaling downstream of PDGFβ.

### Sorafenib blocks PDGFβ-induced hyaluronan production in TED OFs

**Figure 5:**
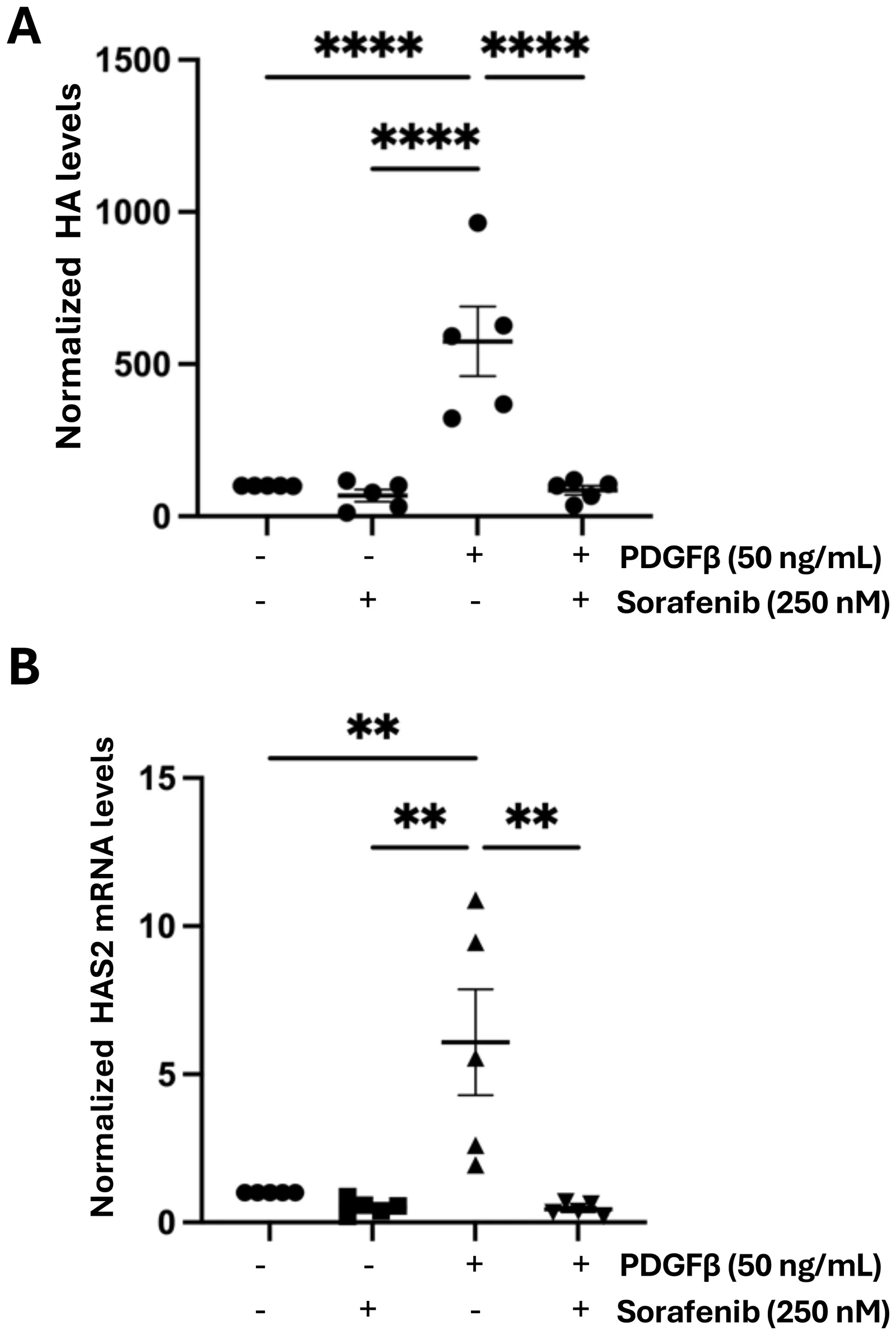
Sorafenib inhibits PDGFβ-induced hyaluronan (HA) and *HAS2* mRNA production in TED orbital fibroblasts. **(A)** Orbital fibroblasts from five TED patient-derived strains were treated with vehicle, PDGFβ (50 ng/mL), or PDGFβ + sorafenib (250 nM) for 48 h. Culture media were collected and HA levels quantified by ELISA. PDGFβ markedly induces HA production, which is inhibited by sorafenib. A summary graph combining normalized HA levels (Veh = 100%) across all strains (each dot represents one strain) shows robust inhibition of HA by sorafenib. Data are mean ± SEM. **(B)** The same cells used in **(A)** were harvested, and total RNA was collected for RT-qPCR. HAS2 mRNA levels were measured and normalized to *18S rRNA* and *TBP*. PDGFβ significantly increased *HAS2 mRNA* expression; co-treatment with sorafenib blunted this effect. Data represent mean ± SEM from n= 5 strains (each data point represents one strain). *HAS1* and *HAS3* mRNA levels were not significantly affected by PDGFβ or sorafenib (data not shown). **p < 0.01, ****p < 0.0001 by one-way ANOVA.

Excessive HA synthesis by OFs contributes to tissue expansion and edema in TED (16, 48, 49). Here, we tested PDGFβ’s ability to promote HA production and whether sorafenib could block HA accumulation. After 48 hours of treatment, PDGFβ (50 ng/mL) strongly induced HA production in all five TED OF strains evaluated, as measured by ELISA (**Figure 5A**). Sorafenib co-treatment (250 nM) reduced HA to near-baseline levels. We also evaluatedsorafenib’s ability to block PDGFβ-induced HA production in a dose-dependent mannerusing 25–500 nM doses of sorafenib in three TED OF strains (**Supplementary Figure 7**). Across three strains, the calculated IC_50_ for sorafenib-mediated inhibition of PDGFβ-induced HA production was ~100 nM, consistent with its inhibition of PDGFβ-AKT signaling.

To determine whether the reduction in HA reflected suppressed HA synthesis rather than reduced cell number, we measured hyaluronan synthase gene expression (**Figure 5B**). PDGFβ significantly induced *HAS2*, the principal hyaluronan synthase in OFs, and sorafenib co-treatment suppressed this induction; the effect was consistent across donor strains. *HAS1* and *HAS3* were not significantly changed by PDGFβ or sorafenib (data not shown). Because *HAS2* expression was normalized to reference genes, the results reflect a direct effect on the HA biosynthetic program rather than cell loss. Sorafenib was used at sub-micromolar concentrations (25 to 500 nM), below the concentrations at which it significantly inhibits fibroblast proliferation (50, 51).

## Discussion

This study provides an integrative transcriptomic and functional analysis directly comparing OFs from patients with TED and non-TED controls. Through unbiased RNA sequencing, we define a fibro-angiogenic phenotype in TED OFs characterized by coordinated upregulation of angiogenic and fibroblast activation programs. Among the most upregulated genes were *FOXC2*, *MET*, *HGF*, *HMGA2*, and *PLEKHG5*, which converge on PI3K/AKT signaling, ECM remodeling, fibroblast activation, and angiogenic activation, while *SFRP2*, a negative regulator of Wnt signaling, was downregulated. This pattern is consistent with prior work demonstrating that PDGF receptors on stromal and perivascular cells drive proliferation and matrix remodeling through targetable signaling pathways (52). Together, these findings implicate the PI3K/AKT pathway as a convergence point that integrates multiple signaling cues to drive OF activation, proliferation, and HA synthesis. Notably, the prominence of angiogenic signatures challenges the established framing of TED as primarily an autoimmune-fibroinflammatory disorder, suggesting that fibrovascular crosstalk may be an underappreciated amplifier of orbital disease.

**Figure 6:**
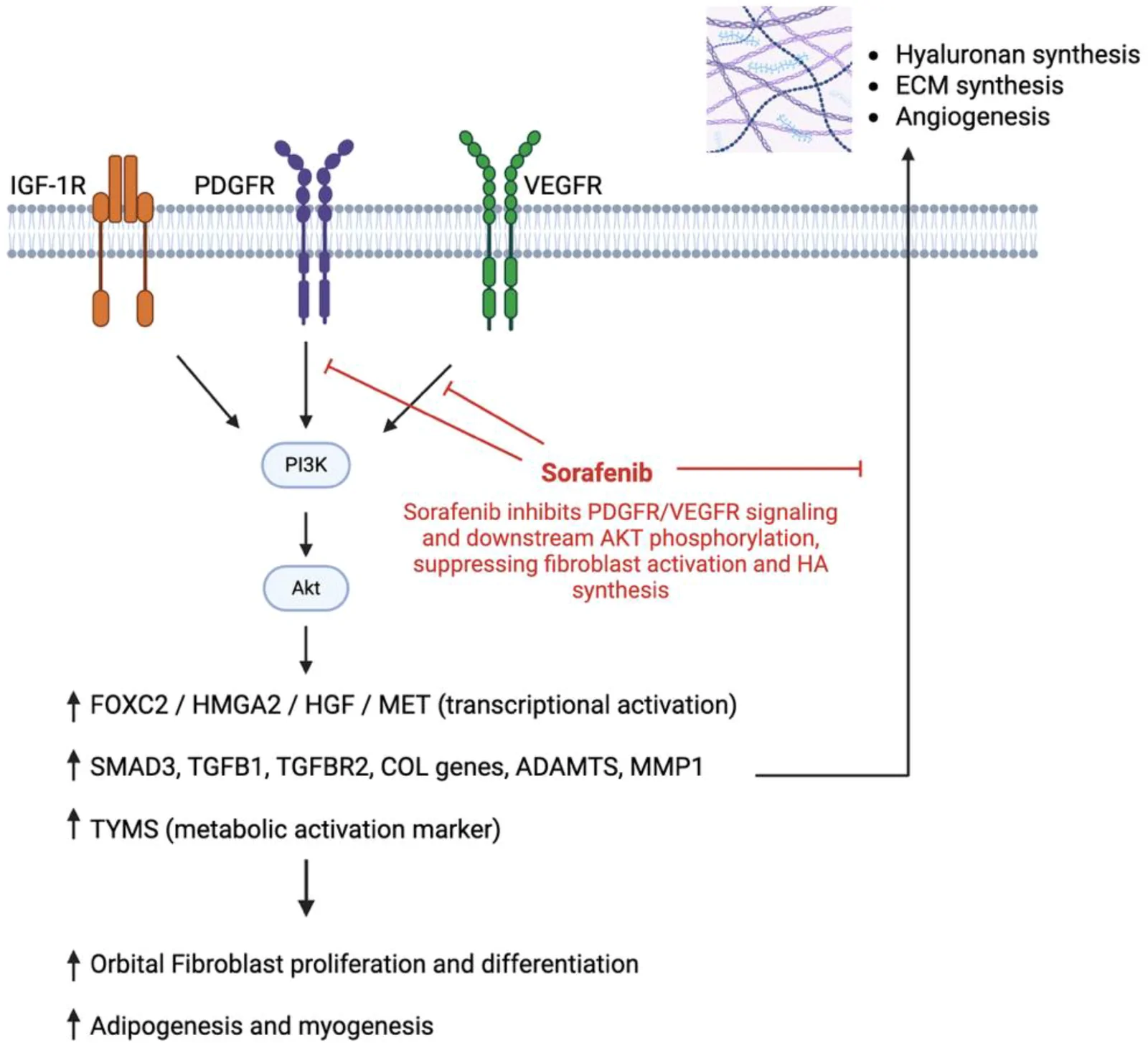
Proposed model illustrating PI3K/AKT and angiogenic pathway activation in TED orbital fibroblasts and sorafenib inhibition. Transcriptomic profiling revealed the activation of PI3K/AKT, ECM remodeling, and angiogenic pathways in TED OFs. PDGFR, VEGFR, and IGF-1R signaling converge on PI3K/AKT to promote the expression of FOXC2, HMGA2, HGF, and collagen-associated genes, thereby enhancing hyaluronan (HA) synthesis and tissue expansion. Sorafenib inhibits VEGFR/PDGFR signaling and downstream AKT activation, thereby suppressing fibroblast activation and extracellular matrix accumulation. Schematic created with BioRender.com.

Building on prior work that identified IGF-1R and TSHR signaling as primary drivers of TED pathogenesis, our findings extend this paradigm by identifying PI3K/AKT as a key downstream node that integrates autoimmune (TSHR), growth factor (IGF-1R, PDGFR), and angiogenic (VEGFR, HGF/MET) responses to coordinate ECM remodeling and tissue expansion. The upregulation of SMAD3, BMP4, and TGFBR2 further links TGFβ/BMP signaling to this network, highlighting crosstalk between growth factor, angiogenic, and fibrotic pathways. This suggests a unified explanation for the diverse clinical manifestations of TED, including fibrosis, adipogenesis, and angiogenesis, within a shared PI3K/AKT-centered signaling network (**Figure 6**).

Our Connectivity Map (LINCS L1000CDS²) analysis identified multiple kinase inhibitors predicted to oppose the TED gene expression profile, including sorafenib, trametinib, and the IGF-1R inhibitor NVP-AEW541, which ranked 5^th^ overall. The identification of an IGF-1R-targeted compound among the top hits is notable: IGF-1R blockade is the mechanism of teprotumumab, the only FDA-approved therapy for TED (10), and its emergence from an unbiased signature-matching approach lends independent pharmacological support to this screen.

Functional studies confirmed that sorafenib, a multi-kinase inhibitor with established activity against Raf kinases, VEGFR, and PDGFRβ (53), dose-dependently suppressed PDGFβ-induced gene expression, AKT phosphorylation, and HA synthesis in TED OFs. Previous studies have revealed similar pathways in other systems; for example, in hepatic stellate cells, PDGFβ-driven activation and survival can be counteracted by sorafenib, in part by modulating Hippo/YAP signaling and inducing ferroptosis (54). Likewise, in both *in vitro* and *in vivo* settings, sorafenib blocked ossification of the posterior longitudinal ligament (OPLL) by disrupting vascularization and osteogenesis through VEGF/PDGF signaling pathways (55). These studies support the concept that PDGF-responsive, matrix-producing stromal cells are a druggable cell type for sorafenib, and our data extend this to OFs in TED.

Sorafenib also offers a therapeutic strategy that complements IGF-1R blockade with teprotumumab, potentially benefiting patients who are refractory to current treatments or relapse after therapy withdrawal. While teprotumumab primarily inhibits IGF-1R-mediated signaling to reduce inflammation and tissue expansion, sorafenib targets PI3K/AKT and angiogenic pathways, addressing additional drivers of fibroblast activation. This dual-pathway approach may enhance therapeutic efficacy and reduce reliance on long-term immunosuppression or invasive surgical interventions, such as orbital decompression, which carry significant risks and morbidity.

While our studies indicate that sorafenib may be effective in treating TED, this work serves as proof of principle for transcriptomic-guided drug repurposing in fibroinflammatory diseases. TED shares core pathogenic components with systemic sclerosis, idiopathic pulmonary fibrosis, and other organ-specific fibrotic diseases (persistent myofibroblast activation, ECM accumulation, growth factor dependence, and angiogenic crosstalk) (2). Sorafenib may also show therapeutic benefit in these diseases. Furthermore, the integrative strategy employed here: RNA-seq → pathway enrichment → LINCS analysis → functional validation could be applied to uncover novel therapeutic targets for a broader spectrum of fibroblast-related disease.

This study also has limitations. The sample size was constrained by access to primary orbital tissue, and we employed bulk RNA-seq, which may mask fibroblast heterogeneity and immune-stromal interactions. Additional single-cell and spatial transcriptomic studies are needed to define TED-enriched fibroblast subsets and determine whether the angiogenic/ECM signature is confined to specific fibroblast subpopulations. *In vitro*, we focused on PDGFβ because it overlapped with the transcriptomic signal detected and with sorafenib’s target profile; however, other RTK ligands present in the orbit (IGF-1, VEGF, HGF/MET, and TGFβ family members) may also sustain the TED phenotype and should be further tested. Finally, sorafenib is a systemic multikinase inhibitor with known dermatologic, gastrointestinal, and metabolic toxicities in differentiated thyroid cancer (56); translating it to TED will require attention to dosing, schedule, and possibly local/topical or depot delivery to minimize off-target effects. Alternatively, more selective multikinase/PI3K inhibitors with reduced adverse profiles may emerge as preferred agents.

Several further limitations bear on the transcriptomic comparison. Sex was imbalanced between groups, with control strains predominantly male (3 of 4) and TED strains predominantly female (6 of 7), resulting in partial sex-disease confounding in this cohort. Unsupervised analysis resolved disease status onto a principal component orthogonal to donor sex, and no sex-chromosome genes appeared among the differentially expressed genes; nonetheless, these analyses are exploratory, and larger, sex-balanced cohorts will be needed to confirm the TED-associated program.

In addition, while sorafenib suppressed PDGFβ-induced expression of genes within the TED signature, we did not perform transcriptome-wide profiling of sorafenib-treated cells. Hence, the extent to which sorafenib reverses the global TED transcriptional state remains to be established. We also did not perform dedicated viability assays; however, the concentrations used here (25 to 500 nM) were well below the micromolar range at which sorafenib inhibits fibroblast proliferation in other fibroblast types (50, 51), and the selective suppression of *HAS2*, with *HAS1* and *HAS3* unchanged, argues against a nonspecific effect on cell number.

In summary, we redefine TED OFs as PI3K/AKT- and angiogenesis-driven orchestrators of TED pathology rather than solely inflammatory/fibrotic targets and demonstrate that an FDA-approved multi-kinase inhibitor can suppress PDGFβ-induced activation of this signaling axis and its downstream fibrotic outputs in TED orbital fibroblasts. The alignment of RNA-seq findings, pathway predictions, and functional validation establishes sorafenib as a plausible candidate for clinical evaluation in TED, particularly for patients with incomplete responses or relapse after IGF-1R blockade. More broadly, this integrated discovery-to-validation pipeline illustrates how transcriptomic profiling can be translated into mechanism-based therapy design across fibroblast-driven disorders.

## Data Availability

The datasets presented in this study can be found in the NCBI Gene Expression Omnibus (GEO) repository, accession number GSE329263.

## Supplementary material

Supplementary Figure 1 Clustering and PCA analysis of TED vs Non-TED OF samples.

Supplementary Figure 2 Expanded heatmap of all differentially expressed genes in non-TED vs TED samples.

Supplementary Figure 3 Uncropped Western blot images for phospho-AKT, AKT, TYMS, and b-Actin.

Supplementary Figure 4 Uncropped Western blot images for phospho-AKT, AKT, TYMS, and b-Actin.

Supplementary Figure 5 Uncropped Western blot images for phospho-AKT, AKT, TYMS, and b-Actin.

Supplementary Figure 6 Uncropped Western blot images for phospho-AKT, AKT, TYMS, and b-Actin.

Supplementary Figure 7 HA ELISA results with sorafenib dose response.

Supplementary Table 1 Complete KEGG (p-adj < 0.05) and Reactome (p-adj <0.10) enrichment results for TED DEGs.

Supplementary Table 2 Complete ranked output of the LINCS L1000CDS2 connectivity analysis (top 50 signatures).

Supplementary Table 3 Expanded donor characteristics for the orbital fibroblast cohort.

Supplementary Table 4 Summary of RNA quality reads and RNA-seq run information for the fibroblast cohort.
