# Supplementary material for: Transcriptomic profiling of thyroid eye disease orbital fibroblasts identifies sorafenib as a novel therapeutic
Source: Front Endocrinol (Lausanne). 2026 Aug 19;17:1870087. doi: 10.3389/fendo.2026.1870087 (PMC13533827; doi:10.3389/fendo.2026.1870087)
Supplement: Supplementary file 1 [file DataSheet1.pdf]

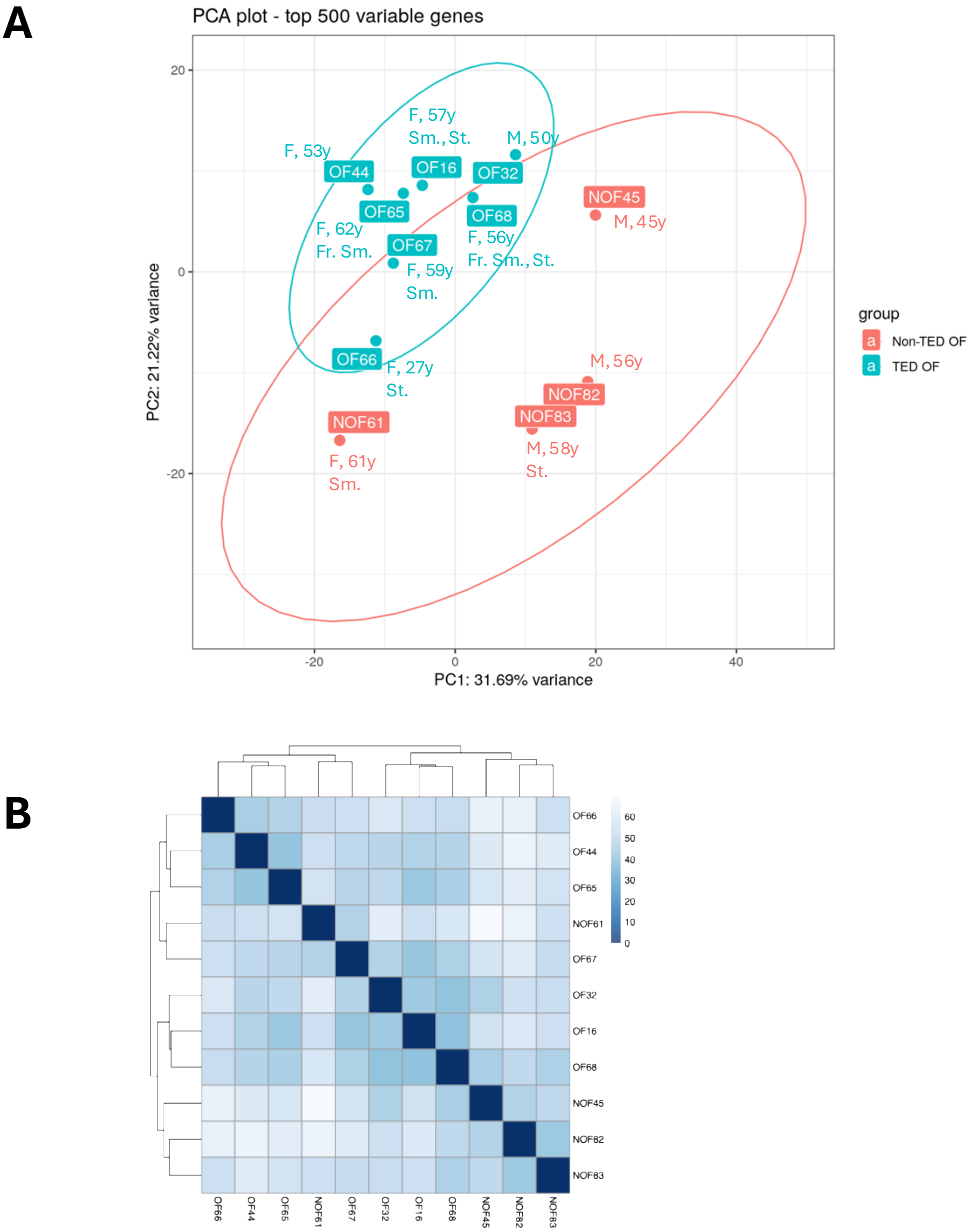

Supplementary Figure 1. Unsupervised analysis of orbital fibroblast transcriptomes and assessment of donor covariates. (A) Principal component analysis of variance-stabilizing transformed (VST) counts using the 500 most variable genes. Each point is one donor strain, colored by group (TED, teal; non-TED, salmon) with 95% confidence ellipses, and annotated with donor covariates: sex (M, male; F, female), age in years, smoking history (Sm, current smoker; Fr Sm, former smoker; unlabeled, never smoker), and prior steroid use (St). PC1 (31.69% of variance) corresponds to donor sex and PC2 (21.22%) to disease status. (B) Unsupervised hierarchical clustering of sample-to-sample Euclidean distances computed on the same VST values (darker indicates greater similarity). Because total transcriptomic distance is dominated by the leading component (donor sex, PC1), samples group partly by sex: the three male control strains (NOF45, NOF82, NOF83) cluster together and the female control (NOF61) groups with the predominantly female TED strains. Disease status is resolved on the orthogonal PC2 (panel A). n = 7 TED and 4 non-TED donor strains.

Supplemental Figure 2

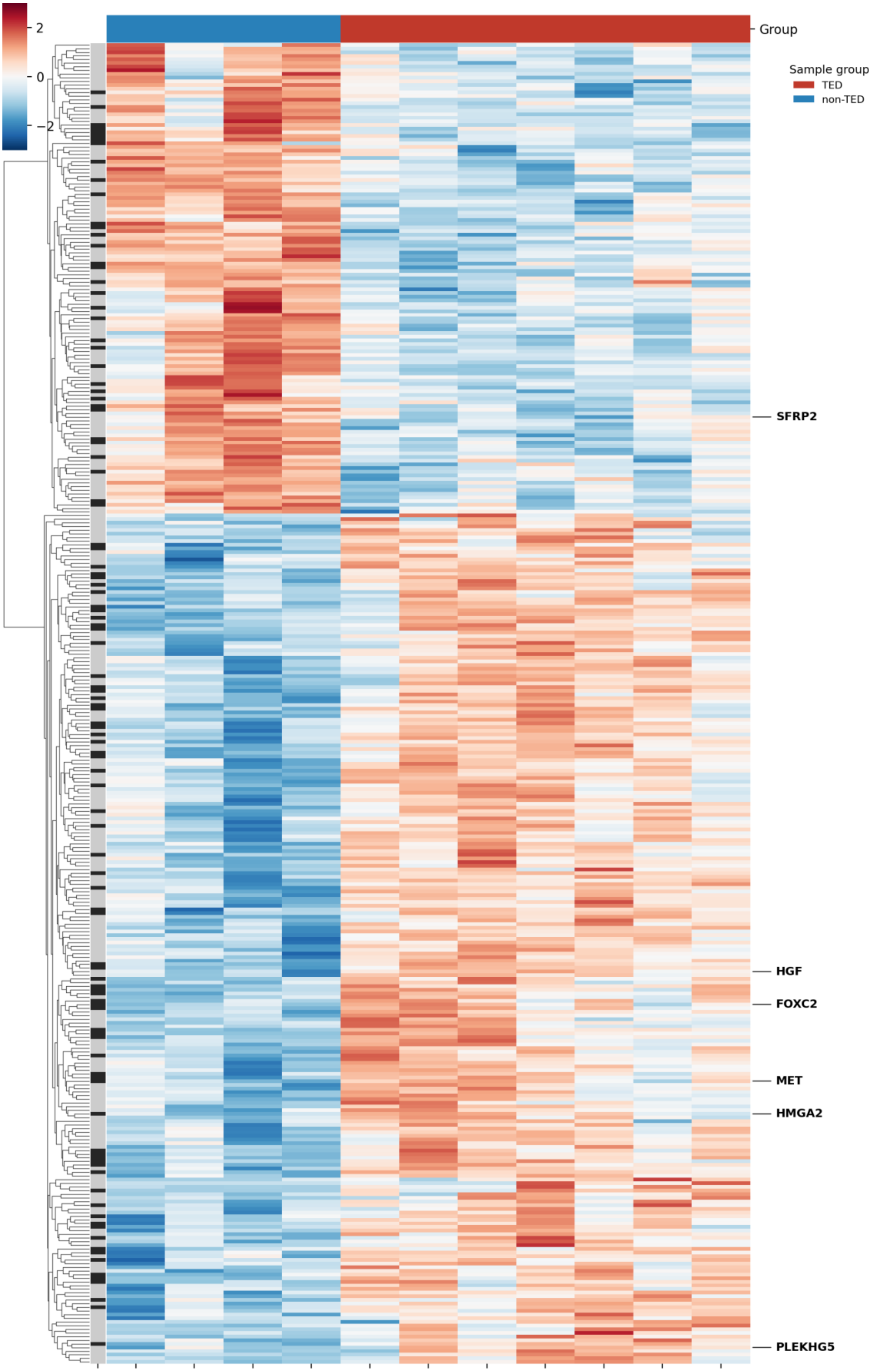

Expanded Heatmap of the differentially expressed genes in TED and non-TED cohorts.

Supplemental Figure 3

Sorafenib Dose-Response (-/+ PDGF) - S080 / S084 TED OF –
phospho-Akt (uncropped blot)

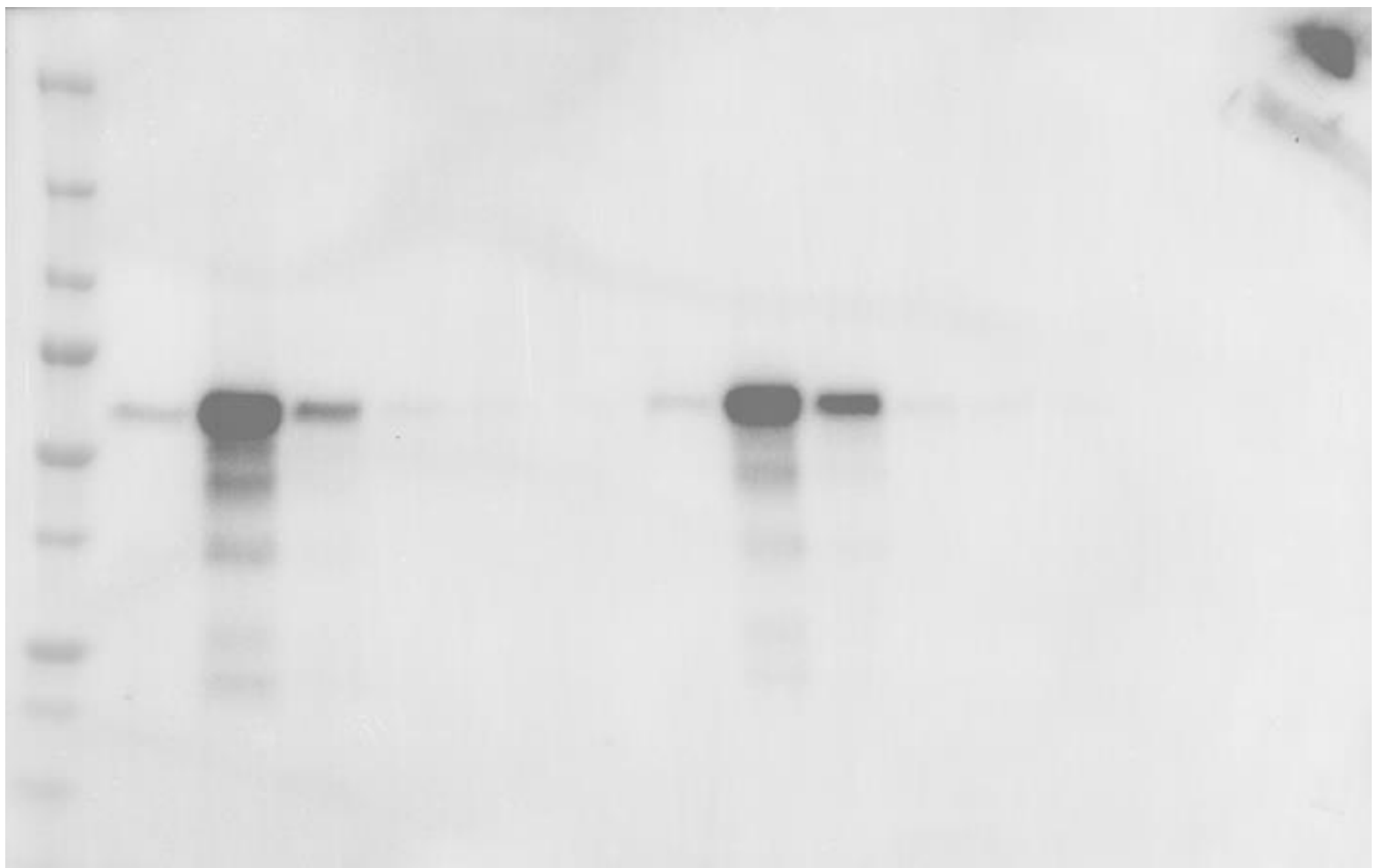

Sorafenib Dose-Response (-/+ PDGF) - S080 / S084 TED OF –
total-Akt (uncropped blot)

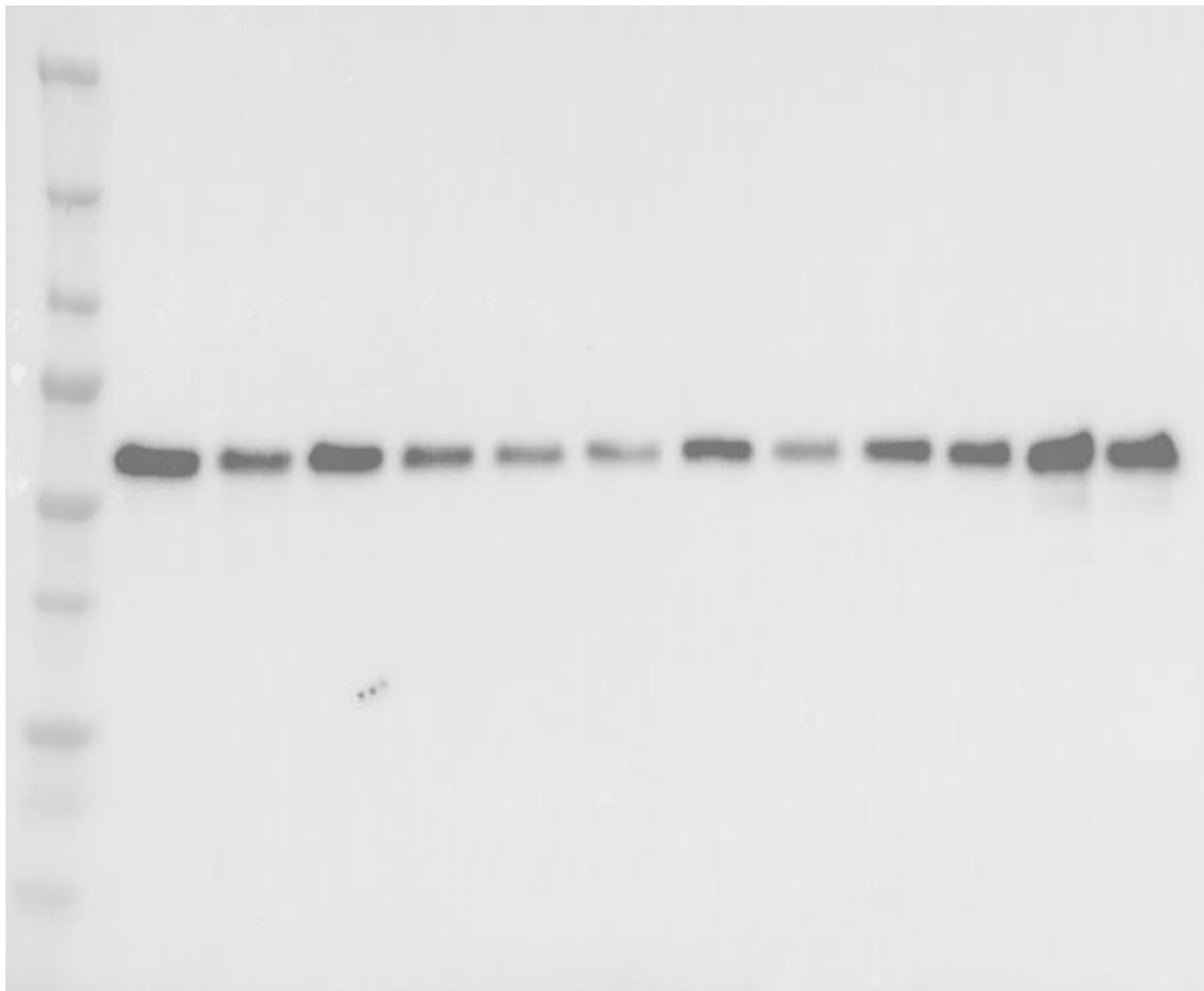

Sorafenib Dose-Response (-/+ PDGF) - S080 / S084 TED OF –
TYMS (uncropped blot)

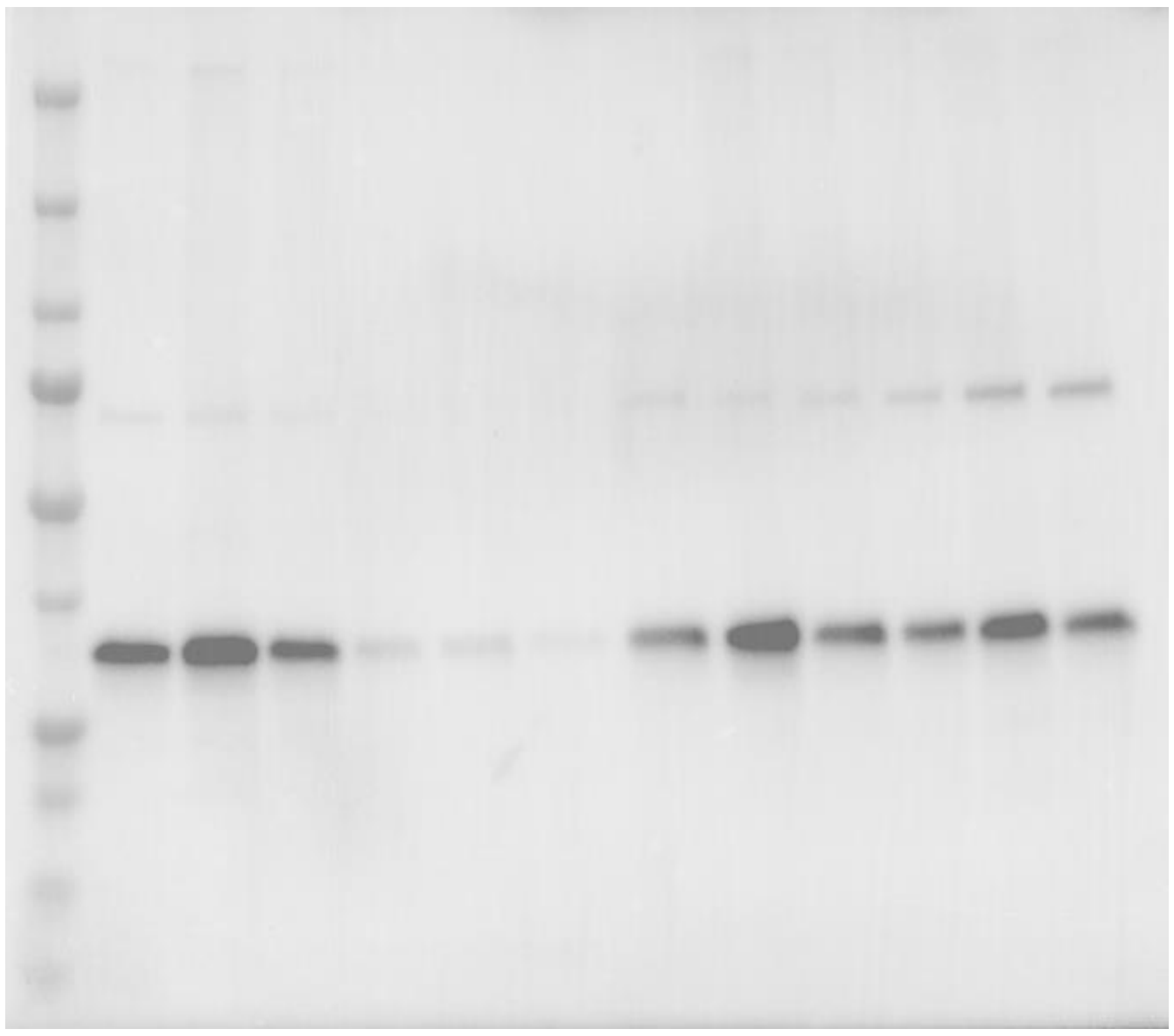

Sorafenib Dose-Response (-/+ PDGF) - S080 / S084 TED OF – **<u>β-Actin</u>** (uncropped blot)

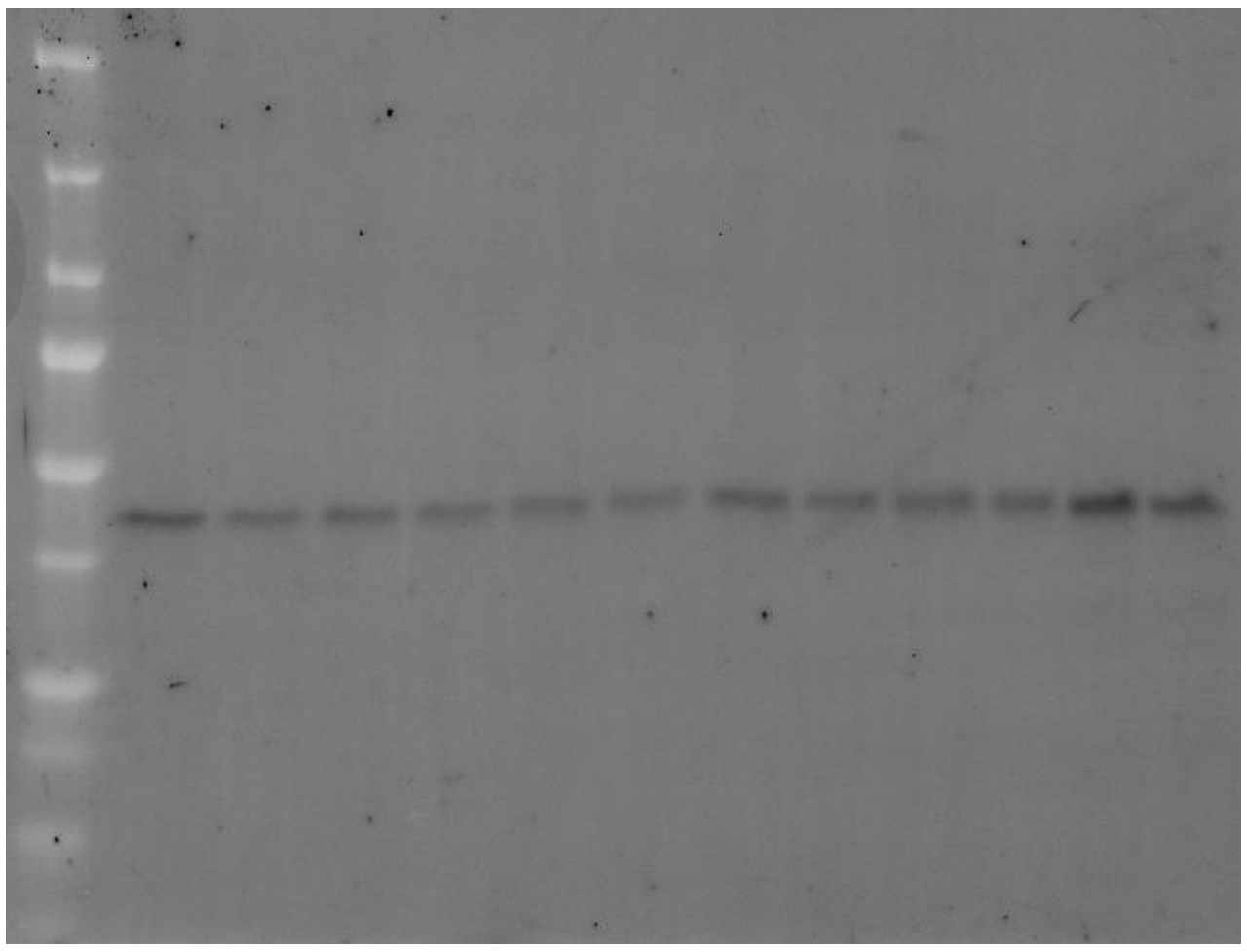

Supplemental Figure 7

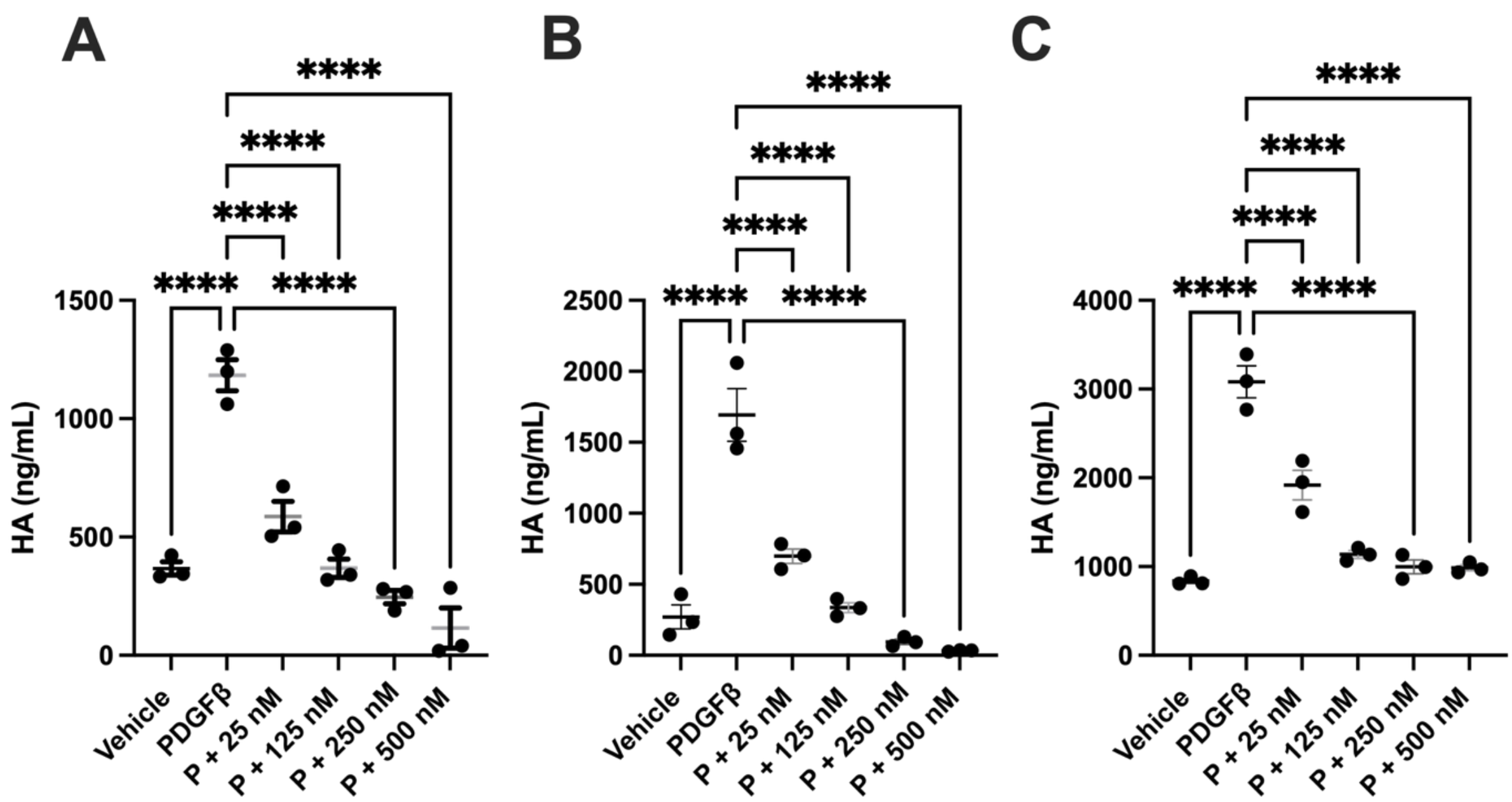

Supplemental Table 1

KEGG p-adj 0.05

Term	P-value	Adjusted p-value	Odds Ratio	Combined Score	Overlap	Genes
Pathways in cancer	1.18E-06	0.000255266	3.740260821	51.06616007	21/531	LAMA5, JUN, CAMK2D, SMAD3, TGFB1, JUP, MMP1, HHIP, HGF, PLEKHG5, PIK3CB, TRAF1, TGFBR2, BMP4, FGF16, SMO, SUFU, COL4A6, RARB, COL4A5, MET
TGF-beta signaling pathway	1.42E-05	0.00153568	8.136847545	90.85223342	8/94	BMP4, SMAD3, TGFB1, PPP2R1A, GDF6, GDF5, TGFBR2, GDF7
Gastric cancer	6.43E-05	0.004651716	5.632748724	54.36618769	9/149	FGF16, SMAD3, TGFB1, JUP, HGF, RARB, PIK3CB, MET, TGFBR2
Rheumatoid arthritis	0.000107749	0.005604077	7.088238321	64.75603465	7/93	IL11, JUN, ATP6V1G2, TGFB1, MMP1, TNFSF11, TLR2
Focal adhesion	0.000129126	0.005604077	4.596060386	41.15643579	10/201	LAMA5, JUN, RELN, HGF, CAPN2, COL4A6, COL4A5, COL9A3, PIK3CB, MET
AGE-RAGE signaling pathway in diabetic complications	0.000169963	0.006146989	6.552383671	56.87423677	7/100	JUN, SMAD3, TGFB1, COL4A6, COL4A5, PIK3CB, TGFBR2
Hippo signaling pathway	0.000664901	0.018391849	4.498810036	32.91271992	8/163	BMP4, SMAD3, TGFB1, PPP2R1A, GDF6, GDF5, GDF7, TGFBR2
Small cell lung cancer	0.000725563	0.018391849	6.048867944	43.72462188	6/92	LAMA5, COL4A6, COL4A5, RARB, PIK3CB, TRAF1
Relaxin signaling pathway	0.000798719	0.018391849	4.987487306	35.5732586	7/129	JUN, TGFB1, MMP1, COL4A6, COL4A5, PIK3CB, TGFBR2
PI3K-Akt signaling pathway	0.000958063	0.018391849	3.084067635	21.43611014	12/354	FGF16, LAMA5, RELN, PPP2R1A, HGF, COL4A6, COL4A5, THEM4, COL9A3, PIK3CB, MET, TLR2
Amoebiasis	0.001244501	0.018391849	5.416024229	36.22789641	6/102	LAMA5, TGFB1, COL4A6, COL4A5, PIK3CB, TLR2
Chagas disease	0.001244501	0.018391849	5.416024229	36.22789641	6/102	JUN, TGFB1, PPP2R1A, PIK3CB, TGFBR2, TLR2
Amphetamine addiction	0.001260235	0.018391849	6.751302083	45.07477846	5/69	CAMK2D, GRIN2A, JUN, FOSB, STX1A
Renal cell carcinoma	0.001260235	0.018391849	6.751302083	45.07477846	5/69	JUN, TGFB1, HGF, PIK3CB, MET
Protein digestion and absorption	0.001308846	0.018391849	5.359916436	35.58239202	6/103	COL13A1, COL25A1, COL4A6, COL4A5, COL9A3, PRSS3
Axon guidance	0.001356081	0.018391849	4.003678161	26.43691287	8/182	SEMA5A, EPHA7, CAMK2D, SMO, LRRC4, PIK3CB, MET, GDF7
Transcriptional misregulation in cancer	0.001900435	0.024258491	3.784154589	23.71027367	8/192	HHEX, JUP, LMO2, HMGA2, PLAT, TRAF1, MET, TGFBR2
Calcium signaling pathway	0.002078174	0.025053545	3.397959184	20.98669821	9/240	FGF16, GRIN2A, HRH1, CAMK2D, MCOLN3, HGF, ADRA1B, MET, MCOLN2
Malaria	0.002716974	0.031030707	7.488513385	44.24390729	4/50	TGFB1, HGF, MET, TLR2
Hepatitis B	0.002951053	0.032018922	3.919040822	22.830738	7/162	JUN, EGR3, SMAD3, TGFB1, PIK3CB, TGFBR2, TLR2
Colorectal cancer	0.003330052	0.034410536	5.329759584	30.40503873	5/86	JUN, SMAD3, TGFB1, PIK3CB, TGFBR2
ECM-receptor interaction	0.003676562	0.035473351	5.200803213	29.15454459	5/88	LAMA5, RELN, COL4A6, COL4A5, COL9A3
Osteoclast differentiation	0.003759848	0.035473351	4.29154986	23.96133936	6/127	JUN, TGFB1, TNFSF11, FOSB, PIK3CB, TGFBR2

Reactome p adj 0.1

Term	P-value	Adjusted P-value	Odds Ratio	Combined Score	Overlap	Genes
Extracellular Matrix Organization	0.000000505	0.000385271	5.058220289	73.33823686	16/300	LAMA5, TGFB1, COL13A1, PCOLCE2, COL25A1, MMP1, ICAM5, LTBP3, GDF5, BMP4, CAPN2, COL4A6, COL4A5, ADAM9, COL9A3, ADAMTS8
Degradation of the Extracellular Matrix	0.00000581	0.002215807	6.773715074	81.66559862	10/140	LAMA5, COL13A1, COL25A1, MMP1, CAPN2, COL4A6, ADAM9, COL4A5, COL9A3, ADAMTS8
Collagen Degradation	0.0000094	0.002391143	10.71029343	123.96768	7/64	COL13A1, COL25A1, MMP1, COL4A6, ADAM9, COL4A5, COL9A3
Signaling by Receptor Tyrosine Kinases	0.0000182	0.003464654	3.305826208	36.0867498	19/536	CYFIP2, LAMA5, ATP6V1G2, EGR3, JUP, HGF, PLAT, PIK3CB, SHB, FGF16, PPP2R1A, POLR2E, FOSB, COL4A5, THEM4, COL9A3, TRIB1, MET, PTPN3
SMAD2 3 Phosphorylation Motif Mutants in Cancer	0.0000304	0.003482629	85.93043478	893.712273	3/6	SMAD3, TGFB1, TGFBR2
TGFBR1 KD Mutants in Cancer	0.0000304	0.003482629	85.93043478	893.712273	3/6	SMAD3, TGFB1, TGFBR2
Signal Transduction	0.000032	0.003482629	1.979103733	20.48632772	53/2613	CYFIP2, HHIP, PLAT, MSI2, PIK3CB, SHB, ADRA1B, GJA1, BAIAP2L1, PPP2R1A, SUFU, ARHGDIB, PIP4K2C, PLEKHG3, DUSP5, ATP6V1G2, HGF, PLEKHG5, TRAF1, TGFBR2, SMO, LINC01139, RARB, COL4A5, TRIB1, MET, GNAZ, LAMA5, RGS17, CAMK2D, CRABP2, LTBP3, FAM169A, ARHGAP22, NEURL1B, HRH1, P2RY6, HCAR1, POLR2E, THEM4, GPC5, JUN, EGR3, SMAD3, TGFB1, JUP, FGF16, PDE10A, DHRS9, FOSB, COL9A3, RGS12, PTPN3
Loss of Function of SMAD2 3 in Cancer	0.0000528	0.004474274	64.44456522	634.7431775	3/7	SMAD3, TGFB1, TGFBR2
Loss of Function of TGFBR1 in Cancer	0.0000528	0.004474274	64.44456522	634.7431775	3/7	SMAD3, TGFB1, TGFBR2
Signaling by TGF-beta Receptor Complex in Cancer	0.0000837	0.006387523	51.55304348	483.9841857	3/8	SMAD3, TGFB1, TGFBR2
Collagen Biosynthesis and Modifying Enzymes	0.000129255	0.008965612	8.538744854	76.45354477	6/67	COL13A1, PCOLCE2, COL25A1, COL4A6, COL4A5, COL9A3
Collagen Chain Trimerization	0.000154143	0.009800909	11.09311741	97.37129714	5/44	COL13A1, COL25A1, COL4A6, COL4A5, COL9A3
Negative Regulation of the PI3K AKT Network	0.000469198	0.027538334	5.484812246	42.038261	7/118	FGF16, PPP2R1A, HGF, THEM4, PIP4K2C, PIK3CB, MET
Collagen Formation	0.000645844	0.035198493	6.193517936	45.49109632	6/90	COL13A1, PCOLCE2, COL25A1, COL4A6, COL4A5, COL9A3
Molecules Associated With Elastic Fibres	0.000876718	0.041808503	10.44541485	73.52866821	4/37	BMP4, TGFB1, LTBP3, GDF5
HSF1-dependent Transactivation	0.000876718	0.041808503	10.44541485	73.52866821	4/37	CAMK2D, HSPA6, COL4A6, TNFRSF21
Germ Layer Formation at Gastrulation	0.000940677	0.042219775	18.40341615	128.2517738	3/17	EOMES, BMP4, SMAD3
MET Activates PTPN11	0.001320486	0.053027926	57.03896104	378.1543745	2/5	HGF, MET
MET Interacts With TNS Proteins	0.001320486	0.053027926	57.03896104	378.1543745	2/5	HGF, MET
Elastic Fibre Formation	0.001691013	0.060924483	8.61441048	54.98085013	4/44	BMP4, TGFB1, LTBP3, GDF5
PI5P, PP2A and IER3 Regulate PI3K AKT Signaling	0.001919451	0.060924483	4.949528005	30.9628411	6/111	FGF16, PPP2R1A, HGF, PIP4K2C, PIK3CB, MET
ECM Proteoglycans	0.001939946	0.060924483	6.083518656	37.99215296	5/76	LAMA5, TGFB1, COL4A6, COL4A5, COL9A3
MET Receptor Activation	0.001965523	0.060924483	42.77705628	266.5864874	2/6	HGF, MET
MET Activates PI3K AKT Signaling	0.001965523	0.060924483	42.77705628	266.5864874	2/6	HGF, MET
Kidney Development	0.001996215	0.060924483	8.203368684	50.99626094	4/46	IRX1, BMP4, FOXC2, IRX2
TGF-beta Receptor Signaling Activates SMADs	0.002162223	0.063452942	8.012186453	49.16772936	4/47	SMAD3, TGFB1, LTBP3, TGFBR2
Long-term Potentiation	0.002326925	0.065757175	12.87847826	78.08488791	3/23	CAMK2D, GRIN2A, NRGN
RUNX3 Regulates CDKN1A Transcription	0.002730626	0.07440955	34.21991342	202.0078317	2/7	SMAD3, TGFB1
PI Metabolism	0.003007686	0.079133244	5.465245392	31.73440875	5/84	PIK3CB, PIP4K2C, PLEKHA4, SYNJ2, ENPP6
Integrin Cell Surface Interactions	0.003165912	0.08016573	5.396655702	31.05944818	5/85	COL13A1, COL4A6, COL4A5, COL9A3, ICAM5
Downregulation of TGF-beta Receptor Signaling	0.003329521	0.08016573	11.19697543	63.87792635	3/26	SMAD3, TGFB1, TGFBR2
Synthesis of PIPs at the Plasma Membrane	0.003362128	0.08016573	7.02896355	40.03122171	4/53	PIK3CB, PIP4K2C, PLEKHA4, SYNJ2
Formation of Intermediate Mesoderm	0.003612934	0.080961121	28.51515152	160.3474045	2/8	BMP4, FOXC2
TGFBR3 Regulates TGF-beta Signaling	0.003612934	0.080961121	28.51515152	160.3474045	2/8	TGFB1, TGFBR2
Attenuation Phase	0.003713813	0.080961121	10.7298913	60.04121203	3/27	HSPA6, COL4A6, TNFRSF21
Activation of HOX Genes During Differentiation	0.004243744	0.087512893	5.018614851	27.4132264	5/91	JUN, POLR2E, HOXB3, RARB, HOXB2
Activation of Anterior HOX Genes in Hindbrain Development During Early Embryogenesis	0.004243744	0.087512893	5.018614851	27.4132264	5/91	JUN, POLR2E, HOXB3, RARB, HOXB2
Signaling by PDGF	0.004656254	0.091277883	6.376516254	34.23898492	4/58	COL4A5, PLAT, COL9A3, PIK3CB
Non-integrin membrane-ECM Interactions	0.004950035	0.091277883	6.260262009	33.23172804	4/59	LAMA5, TGFB1, COL4A6, COL4A5
HSF1 Activation	0.005024471	0.091277883	9.536231884	50.47942531	3/30	HSPA6, COL4A6, TNFRSF21
Laminin Interactions	0.005024471	0.091277883	9.536231884	50.47942531	3/30	LAMA5, COL4A6, COL4A5
MET Activates PTK2 Signaling	0.005024471	0.091277883	9.536231884	50.47942531	3/30	LAMA5, HGF, MET
Nuclear Events (Kinase and Transcription Factor Activation)	0.005574371	0.096951342	6.039990807	31.34499046	4/61	EGR3, PPP2R1A, FOSB, TRIB1
POU5F1 (OCT4), SOX2, NANOG Repress Genes Related to Differentiation	0.005717969	0.096951342	21.38419913	110.4310322	2/10	EOMES, HHEX
MET Receptor Recycling	0.005717969	0.096951342	21.38419913	110.4310322	2/10	HGF, MET
Developmental Biology	0.005976373	0.09833932	1.776752649	9.096869468	27/1385	SEMA5A, FOXC2, PIK3CB, HHEX, RELN, PERP, POLR2E, EOMES, IRX1, IRX2, EPHA7, JUN, MYOCD, SMAD3, TGFB1, JUP, CNTN6, TBX2, BMP4, PKP2, HOXB3, RARB, COL4A5, HOXB2, COL9A3, MET, STX1A
VEGFA-VEGFR2 Pathway	0.006057599	0.09833932	4.589632325	23.43669018	5/99	CYFIP2, JUP, THEM4, PIK3CB, SHB

Supplementary Table 2. Complete ranked output of the LINCS L1000CDS² connectivity analysis (top 50 signatures).

Rank	Score (1 − cos α)	Perturbation	Target	Mechanism of action
1	1.5737	Brefeldin A	Protein transport	ER/Golgi transport inhibitor
2	1.5289	89671	Protein transport	ER/Golgi transport inhibitor
3	1.5277	BRD-K94390040	Unknown	Uncharacterized
4	1.5202	BRD-K08547377	Unknown	Uncharacterized
5	1.5112	NVP-AEW541	IGF-1R	IGF-1R inhibitor
6	1.5055	BRD-A50737080	Unknown	Uncharacterized
7	1.5051	XMD16-144	Kinase (general)	Kinase inhibitor probe
8	1.5038	TPCA-1	IKK-2	IKK-2 inhibitor
9	1.4989	Trametinib	MEK1 / MEK2	MEK1/2 inhibitor
10	1.4977	VX-680	Aurora kinase	Aurora kinase inhibitor
11	1.4966	Afatinib	EGFR / HER2	EGFR/HER2 inhibitor
12	1.4951	HY-10992	Chk1 / Chk2	Checkpoint kinase inhibitor
13	1.4931	BRD-U32365159	Kinase (general)	Kinase inhibitor probe
14	1.4929	CPT-11	Topoisomerase I	Topoisomerase I inhibitor
15	1.4925	Sorafenib	RAF, VEGFR, PDGFR, RET	Multi-kinase inhibitor
16	1.4915	Dasatinib	BCR-ABL, SRC, PDGFR, KIT	Multi-kinase tyrosine inhibitor
17	1.4889	TG101348	JAK2	JAK2 inhibitor
18	1.4876	BRD-A76490030	EGFR (predicted)	Predicted EGFR inhibitor
19	1.4841	NVP-TAE684	ALK	ALK inhibitor
20	1.4802	Bithionate sodium	Parasitic enzymes	Antiparasitic agent
21	1.4795	XMD16-144	Kinase (general)	Kinase inhibitor probe
22	1.4770	GSK-1070916	Aurora B / Aurora C	Aurora kinase inhibitor
23	1.4756	Brefeldin A	Protein transport	ER/Golgi transport inhibitor
24	1.4750	BRD-K22828860	Unknown	Uncharacterized
25	1.4747	Tpl2 (COT) inhibitor	MAP3K8	Tpl2 kinase inhibitor
26	1.4732	BRD-A17065207	Unknown	Uncharacterized
27	1.4729	BRD-A36630025	Unknown	Uncharacterized
28	1.4724	Geldanamycin	HSP90	HSP90 inhibitor
29	1.4723	Selumetinib	MEK1 / MEK2	MEK1/2 inhibitor
30	1.4722	BRD-K18163752	Unknown	Uncharacterized
31	1.4716	Eburnamonine (-)	Tubulin / vasculature	Vinca alkaloid derivative
32	1.4700	Selumetinib	MEK1 / MEK2	MEK1/2 inhibitor
33	1.4686	Ibrutinib	BTK	BTK inhibitor
34	1.4680	BRD-K32827536	Unknown	Uncharacterized
35	1.4677	BRD-A17065207	Unknown	Uncharacterized
36	1.4662	BRD-A25088322	SUMO	SUMOylation pathway inhibitor
37	1.4654	Dovitinib	FGFR, VEGFR, PDGFR	Multi-kinase tyrosine inhibitor
38	1.4643	EI-346	Unknown	Uncharacterized
39	1.4641	AZD-7762	Chk1	CHK1 inhibitor
40	1.4640	BRD-K28366633	Unknown	Uncharacterized
41	1.4638	Na-Tosyl-Phe chloromethyl ketone	Serine proteases	Serine protease inhibitor
42	1.4636	BRD-K54256913	Unknown	Uncharacterized
43	1.4636	BRD-K82732294	Unknown	Uncharacterized
44	1.4627	Ethacrynic acid	NKCC2 co-transporter	Loop diuretic
45	1.4626	XMD-885	Kinase (general)	Kinase inhibitor probe
46	1.4619	PD-0325901	MEK1 / MEK2	MEK1/2 inhibitor
47	1.4597	Canertinib	EGFR	EGFR inhibitor
48	1.4596	FIT	Unknown	Uncharacterized
49	1.4592	BRD-K24632213	Unknown	Uncharacterized
50	1.4591	NCGC00182371-01	Unknown	Uncharacterized

Full L1000CDS² Table. Signatures are ranked by connectivity score (1 − cosine similarity); higher scores indicate stronger predicted reversal of the input signature. Scores across the top 50 span a narrow range (1.4591 to 1.5737). Sorafenib ranked 15th (1.4925), within 5.2% of the top-scoring perturbation. Molecules may appear more than once, reflecting independent L1000 signatures.

Supplemental Table 3

Strain	Group	Sex	Age (y)	Smoking	Prior steroid	Surgical indication
TED_1	TED	F	57	Current	Yes	Orbital decompression
TED_2	TED	M	50	Never	No	Orbital decompression
TED_3	TED	F	53	Never	No	Orbital decompression
TED_4	TED	F	62	Former	No	Orbital decompression
TED_5	TED	F	27	Never	Yes	Orbital decompression
TED_6	TED	F	59	Current	No	Orbital decompression
TED_7	TED	F	56	Former	Yes	Orbital decompression
Non-TED_1	Non-TED	M	45	Never	No	Orbital fracture repair
Non-TED_2	Non-TED	F	61	Current	No	Orbital fracture repair
Non-TED_3	Non-TED	M	56	Never	No	Orbital fracture repair
Non-TED_4	Non-TED	M	58	Never	Yes	Orbital fracture repair

Supplemental Table 3: Expanded donor characteristics for the orbital fibroblast cohort.

Supplemental Table 4

Sample	RIN	Reads (M)	Uniquely mapped (%)	Assigned (%)	Duplication (%)	GC (%)
TED_1	10	33.3	92.2	69.5	61.5	49.3
TED_2	10	32.6	91.8	69.4	61.7	50.0
TED_3	10	33.4	93.3	74.6	61.4	49.5
TED_4	10	35.8	91.7	68.9	62.8	49.1
TED_5	10	28.2	93.2	73.8	60.0	49.8
TED_6	10	32.3	92.8	73.0	61.4	49.7
TED_7	10	31.2	92.0	70.7	62.2	49.7
Non-TED_1	10	32.7	92.5	72.4	63.6	50.1
Non-TED_2	10	33.1	93.3	74.7	60.9	48.9
Non-TED_3	10	33.2	91.8	69.7	65.3	50.5
Non-TED_4	10	32.9	91.4	69.0	63.5	49.9

Supplemental Table 4 Summary of RNA quality, reads and RNAseq run information for the fibroblast cohort. Per-sample sequencing quality metrics. RIN, RNA integrity number. Reads, reads passing filter (fastp 0.23.1). Uniquely mapped, STAR 2.7.9a uniquely mapped reads as a percentage of input reads (aligned to GRCh38.p13, GENCODE release 42). Assigned, reads assigned to genes by featureCounts (Subread 2.0.1, -s 2). Duplication and GC content from FastQC/fastp.

Cohort summary: RIN = 10 for all samples; reads mean 32.6M (range 28.2-35.8); uniquely mapped mean 92.4% (range 91.4-93.3); assigned mean 71.4% (range 68.9-74.7); duplication mean 62.2% (range 60.0-65.3); GC mean 49.8% (range 48.9-50.5).
